# The Q/R editing site of AMPA receptor GluA2 subunit acts as an epigenetic switch regulating dendritic spines, neurodegeneration and cognitive deficits in Alzheimer’s disease

**DOI:** 10.1186/s13024-023-00632-5

**Published:** 2023-09-28

**Authors:** Amanda L. Wright, Lyndsey M. Konen, Bruce G. Mockett, Gary P. Morris, Anurag Singh, Lisseth Estefania Burbano, Luke Milham, Monica Hoang, Raphael Zinn, Rose Chesworth, Richard P. Tan, Gordon A. Royle, Ian Clark, Steven Petrou, Wickliffe C. Abraham, Bryce Vissel

**Affiliations:** 1 St Vincent’s Clinical School, St Vincent’s Hospital Sydney, Faculty of Medicine, University of New South Wales, Darlinghurst, NSW 2010 Australia; 2 School of Rural Medicine, Charles Sturt University, Orange, NSW 2800 Australia; 3 Centre for Neuroscience and Regenerative Medicine, St Vincent’s Centre for Applied Medical Research, St Vincent’s Hospital Sydney, Darlinghurst, NSW 2010 Australia; 4 Department of Psychology, Brain Health Research Centre, Brain Research New Zealand, University of Otago, Box 56, Dunedin, 9054 New Zealand; 5 Tasmanian School of Medicine, College of Health and Medicine, University of Tasmania, Hobart, TAS 7005 Australia; 6 Florey Institute of Neuroscience and Mental Health, University of Melbourne, Parkville, VIC 3010 Australia; 7 Department of Anatomy and Neuroscience, University of Melbourne, Parkville, VIC 3010 Australia; 8 School of Pharmacy, University of Waterloo, Kitchener, ON N2G 1C5 Canada; 9 School of Medicine, Western Sydney University, Campbelltown, NSW 2560 Australia; 10 Chronic Diseases, School of Medical Sciences, Faculty of Health and Medicine, University of Sydney, Sydney, NSW 2050 Australia; 11 Charles Perkins Centre, University of Sydney, Sydney, NSW 2006 Australia; 12 Middlemore Hospital, Counties Manukau DHB, Otahuhu, Auckland, 1062 New Zealand; 13 Faculty of Medical and Health Sciences, University of Auckland, Grafton, Auckland, 1023 New Zealand; 14 Research School of Biology, Australian National University, Canberra, ACT 0200 Australia

**Keywords:** RNA editing, GluA2, Alzheimer’s disease, AMPAR, Neurodegeneration

## Abstract

**Background:**

RNA editing at the Q/R site of GluA2 occurs with ~99% efficiency in the healthy brain, so that the majority of AMPARs contain GluA2(R) instead of the exonically encoded GluA2(Q). Reduced Q/R site editing increases AMPA receptor calcium permeability and leads to dendritic spine loss, neurodegeneration, seizures and learning impairments. Furthermore, GluA2 Q/R site editing is impaired in Alzheimer’s disease (AD), raising the possibility that unedited GluA2(Q)-containing AMPARs contribute to synapse loss and neurodegeneration in AD. If true, then inhibiting expression of unedited GluA2(Q), while maintaining expression of GluA2(R), may be a novel strategy of preventing synapse loss and neurodegeneration in AD.

**Methods:**

We engineered mice with the ‘edited’ arginine codon (CGG) in place of the unedited glutamine codon (CAG) at position 607 of the *Gria2* gene. We crossbred this line with the J20 mouse model of AD and conducted anatomical, electrophysiological and behavioural assays to determine the impact of eliminating unedited GluA2(Q) expression on AD-related phenotypes.

**Results:**

Eliminating unedited GluA2(Q) expression in AD mice prevented dendritic spine loss and hippocampal CA1 neurodegeneration as well as improved working and reference memory in the radial arm maze. These phenotypes were improved independently of Aβ pathology and ongoing seizure susceptibility. Surprisingly, our data also revealed increased spine density in non-AD mice with exonically encoded GluA2(R) as compared to their wild-type littermates, suggesting an unexpected and previously unknown role for unedited GluA2(Q) in regulating dendritic spines.

**Conclusion:**

The Q/R editing site of the AMPA receptor subunit GluA2 may act as an epigenetic switch that regulates dendritic spines, neurodegeneration and memory deficits in AD.

**Supplementary Information:**

The online version contains supplementary material available at 10.1186/s13024-023-00632-5.

## Background

The core clinical feature of Alzheimer’s disease (AD) dementia, differentiating AD from other memory disorders [1], is considered to be the presence of a hippocampal-type amnestic syndrome [2, 3]. Alongside this syndrome, AD is identified as a neurodegenerative disorder due to long-standing evidence of neuron loss in specific brain regions [4–9], with the magnitude of atrophy correlating with cognition [6, 10–13]. Furthermore, synapse alterations and/or loss in multiple brain regions involved in memory (the hippocampus, cingulate cortex and temporal cortex) are strongly correlated to cognitive decline in AD [14–16] and synapse dysfunction could precede neuronal pathology [17, 18].

Although the causes of synapse and neuronal pathology in AD are unknown, correlative studies have provided several hypotheses. Two proteins, amyloid-β (Aβ) and tau, both of which can aggregate into soluble oligomers and insoluble plaques and tangles, respectively, have long been hypothesised to be toxic triggers of synapse and neuronal loss [17, 19]. However, it is not yet clear if these proteins are representative of a specific dementia phenotype, or if/how they are mechanistically involved in driving synaptic and neuronal degeneration [17]. Additionally, there are many genetic and non-genetic risk factors potentially contributing to synapse and neuronal pathology in AD, beyond Aβ and tau [17]. Of importance to the current work, multiple studies have identified that dysfunctional RNA processing mechanisms could potentially contribute to disease [20–24].

RNA undergoes > 100 modifications (collectively termed the ‘epitranscriptome’, for recent reviews see: [25–27]), which can physiologically diversify the transcriptome. In one such modification, RNA ‘editing’, nucleotides can be inserted, deleted or modified in an RNA transcript, thereby potentially altering the amino acid code and subsequent function of a translated protein [28]. Adenosine-to-inosine RNA editing (the most common editing event, with the inosine being interpreted as a guanosine) occurs at millions of genomic sites in humans [29]. Notably, the brain is the most edited tissue [30, 31] and editing sites in coding regions are enriched in neural genes [32–34]. RNA editing is a physiologically important process, as evidenced by the lethal phenotype, or change in memory function, of mice with a knock-out (KO) of the editing enzymes adenosine deaminases acting on RNA 1, 2 and 3 (ADAR1, ADAR2 and ADAR3 [35–37]), which are highly expressed in the brain [30]. Furthermore, studies have identified hyper- and hypo- editing of many RNAs as well as dysregulation of the editing enzymes under a variety of degenerative and hyperexcitable conditions [38–47], including AD [7, 35, 48–53]. These findings have raised the possibility that ADAR2 downregulation and/or dysfunctional RNA editing could be causally related to these conditions.

One conserved [54] and well-studied editing site is the ‘Q/R’ site of GluA2, a protein subunit of the tetrameric α-amino-3-hydroxy-5-methyl-4-isoxazolepropionic acid receptor (AMPAR). ADAR2-mediated editing at this site converts an exonically encoded CAG codon for glutamine (Q) to a CIG codon that is recognised during translation as a CGG codon for arginine (R). The Q/R site of GluA2 is ~99% edited in the healthy adult brain [55], however, decreases in the editing efficiency of this site in genetically-modified mice lead to seizures, neurodegeneration and early lethality [56–58]. This is thought to be because unedited GluA2(Q)-containing AMPARs have a higher conductance and are Ca^2+^-permeable. Thus, it is possible that a pathological increase in the amount of unedited GluA2(Q) could cause synapse and neuron dysfunction and loss through excitotoxicity, a theory that ties in neatly with the hypothesis that calcium dyshomeostasis is aetiologically involved in AD [59].

Dysregulation in Q/R site GluA2 editing has been implicated in neurodegeneration in motor neuron disease (MND) [60] and ischemia [44, 61]. Furthermore, deficient GluA2 Q/R site editing has also been observed in the prefrontal cortex [51], temporal lobe [52] and hippocampus [21, 50] of human AD patients. However, deficits were not seen in aged mice [62], or in one AD mouse model [63], possibly because they are below the level of detection with the methods used. This has ignited controversy over the hypothesis that unedited GluA2(Q) contributes to neurodegeneration [64]. Despite the growing evidence that RNA editing deficits are linked to neurodegenerative diseases, there is a distinct lack of in vivo evidence directly linking RNA editing deficits to the emergence of human AD-related pathology and cognitive dysfunction.

In this study, we provide the first proof-of-principle that eliminating GluA2(Q) expression can improve several phenotypes in a mouse model of AD. We created mice with a point mutation (A➔G) at the Q/R site of GluA2, thus negating the need for editing at this site and ensuring all GluA2-containing AMPARs are in the edited GluA2(R) form and impermeable to Ca^2+^. We crossed this line with the J20 mouse model of AD, which we have previously shown develops neurodegeneration, plaque deposition, inflammation and behavioural deficits [65]. We report that exonically encoding GluA2(R) prevents dendritic spine and neuron loss, synaptic dysfunction and memory impairments without affecting Aβ-pathology. Additionally, our data revealed encoding GluA2(R) in healthy wildtype (WT) mice led to increased hippocampal spine density, indicating a previously undiscovered role for GluA2(Q) in regulating dendritic spines. Our findings suggest that the Q/R editing site of the AMPA receptor GluA2 subunit acts as an epigenetic switch regulating dendritic spines and through which hippocampal synaptic and neuronal degeneration occurs in AD.

## Materials and methods

### Animals

All animal experiments were performed with the approval of the Garvan Institute and St. Vincent’s Hospital Animal Ethics Committee (protocol IDs AEC 08/20, 11/51, 14/40 and 17/28) as well as the animal ethics committee of the Florey Institute of Neuroscience and Mental Health (for ECoG recordings; protocol ID AEC 14-025) and the animal ethics committee of Western Sydney University (for tissue collection from 5xFAD mice; protocol ID A13397), in accordance with National Health and Medical Research Council animal experimentation guidelines and the Australian Code of Practice for the Care and Use of Animals for Scientific Purposes (2013). Animals used in electrophysiology experiments were performed with the approval of the University of Otago Animal Ethics Committee (ET10-15). For all studies, only male mice were used. Mice were housed in individually ventilated cages (IVC) and maintained on a 12 h light/dark cycle (lights on at 7:00am).

#### 5xFAD mice

Note: these mice were only used for Sup Fig. 5b. Hemizygous and non-transgenic littermates were from the B6SJL-Tg(APPSwFlLon,PSEN1*M146L*L286V)6799Vas/Mmjax (aka: 5xFAD) line (MMRRC stock # 34840). Transgenic mice express human amyloid precursor protein (hAPP) containing the Swedish, Florida and London familial Alzheimer’s disease mutations as well as the Presenilin 1 (PS1) gene with two mutations. Both genes are regulated by the mouse *Thy1* promoter [66]. Mice were maintained on a mixed background by breeding within the colony and were housed at a maximum of three per cage. Genotyping was performed through PCR amplification of genomic DNA using standard practices and using primer sequences recommended by The Jackson Laboratory.

#### J20 mice

Hemizygous transgenic and non-transgenic littermates were from the B6.Cg-Tg(PDGFB-APPSwInd)20Lms/2J (aka: J20) line (MMRRC stock #34836), which express hAPP containing both the Swedish and Indiana mutations, under a *PDGF-β* promoter [67]. Mice were maintained on a C57BL/6J background and were housed at a maximum of five per cage. Genotyping was performed through PCR amplification of genomic DNA using standard practices and using primer sequences recommended by The Jackson Laboratory.

#### *Gria2*^tm1BViss^ mice

To develop the *Gria2*^tm1BViss^ mice (i.e. exonically encoded GluA2(R)), a construct was generated from DNA cloned from a 129 SvEv DNA genomic library (Sup Fig. 1A). A neomycin gene, flanked by loxP sites, was placed downstream of exon 11. In addition, a single point adenosine to guanine mutation was made at the Q/R editing site of intron 11 (C**A**G ➔ C**G**G). The construct was electroporated into CCE embryonic stem (ES) cells, which were derived from 129SvEv mice. Colonies resistant to G418 were isolated and an ES cell colony that contained the allele was identified. This ES cell colony was electroporated with Cre-expressing plasmid and re-plated in the absence of G418, thus excising the neomycin and leaving a single loxP site in addition to the point mutation. Genotyping was performed through PCR amplification of genomic DNA using standard practices. Specific oligonucleotide primers for the *Gria2* wild-type allele were: common forward- 5′-GTG TCT CTT GGG GAA GTT CAA T-3′; and reverse- 5′- TGA TAT ATT TCC CTC TTC TCA GCC AGT GG -3′. For the targeted allele, a reverse primer was designed from within the loxP sequence as follows: reverse- 5′-TGC CCA CAT CTA AGA TTG TTG GAC-3′. Additionally, presence of only the targeted Gria2^G/G^ transcript (aka: edited GluA2(R)) was confirmed via RT-PCR of the *Gria2* gene containing the Q/R site and subsequent digestion with the *BbvI* restriction enzyme as previously described [58].

#### *Gria2*^tm1BViss^ x J20 cross

*Gria2*^tm1BViss^ mice were maintained on a C57BL/6J background for at least 10 generations before being crossed with hemizygous J20 mice. To obtain littermates of all possible genotypes, the bi-transgenic colony was maintained by mating female hemizygous *Gria2*^tm1BViss^ mice with double hemizygotes from the cross colony. Of the resulting possible genotypes, four were assessed (see Table 1 below). Mice were housed at a maximum of five per cage. For behavioral studies that involved the radial arm maze (RAM), mice were housed individually. Food and water were available *ad libitum* unless dietary restrictions were required for mice undergoing RAM testing.

**Table 1:** Description of genotypes used in study. All mice are littermates from crossing *Gria2*^tm1BViss^ mice with J20 mice

**Genotype**	**Genotype Description**
WT	Wildtype for both transgenes
Gria2^G/G^	Homozygous for Gria2 targeted allele (aka: edited GluA2(R)) and wildtype for J20
J20	Wildtype for Gria2 and hemizygous for J20
Gria2^G/G^/J20	Homozygous for Gria2 targeted allele (aka: edited GluA2(R)) and hemizygous for J20

### Electrophysiology

#### Field potential electrophysiology

All recordings were made by an experimenter blind to the genotype of each mouse. Mice (33–54 weeks of age), were deeply anaesthetised with ketamine (100 mg/kg, i.p.), and the brains removed and chilled in ice-cold and oxygenated modified artificial cerebrospinal fluid (aCSF) in which sucrose was substituted for NaCl (composition in mM: sucrose 210, glucose 20, KCl 2.5, NaH_2_PO_4_ 1.25, NaHCO_3_ 26, CaCl_2_ 0.5, MgCl_2_ 3, pH 7.4 when gassed with 95% O_2_-5% CO_2_). Whole hemisphere parasagittal slices (400 µm) containing transverse sections of the dorsal hippocampus were cut in a manner similar to that described previously [68] using a vibratome (VT1000, Leica Microsystems). Slices were transferred to a custom-built incubation chamber containing aCSF (mM: 124 NaCl, 3.2 KCl, 1.25 NaH_2_PO_4_, 26 NaHCO_3_, 2.5 CaCl_2_, 1.3 MgCl_2_, 10 D-glucose) bubbled with carbogen. Slices were incubated at interface for 30 min at 32 °C and then at room temperature for at least 90 min. After this recovery period, they were transferred to a recording chamber at 32.5 °C and superfused (2 mL/min) with oxygenated aCSF.

Baseline field excitatory postsynaptic potentials (fEPSPs) in area CA1 of the hippocampus were elicited by stimulation of the Schaffer collateral-commissural pathway at 0.017 Hz (diphasic pulses, 0.1 ms half-wave duration) using a Teflon-coated 50 μm tungsten wire monopolar electrode (A-M Systems Inc). Evoked responses were recorded using a glass microelectrode filled with aCSF (2–3 MΩ) and placed in stratum radiatum. During periods of baseline recording, the stimulation intensity was adjusted to elicit a fEPSP with an initial slope value of 40% of the maximum slope elicited when delivering 200 µA of current. Drugs were bath-applied by switching to an identical preheated and oxygenated aCSF solution that contained the compound of interest.

Once fEPSPs were established, we conducted three electrophysiological procedures in stratum radiatum. These were: paired-pulse facilitation (PPF) to assess presynaptic short-term plasticity; an input-output test to assess basal synaptic transmission; and induction of long-term potentiation (LTP) to assess synaptic plasticity. PPF was induced in stratum radiatum by delivering paired stimuli (3 pairs at 10 s intervals, 1 mV initial response) at interpulse intervals ranging from 20–200 ms in the presence of D-AP5 (50 µM, Tocris). PPF was expressed as a ratio of the response amplitudes and was calculated as EPSP 2 amplitude/EPSP 1 amplitude. Basal synaptic transmission was determined across stimuli of increasing intensity ranging from 10–200 µA (average slope of 3 responses at each stimulus intensity) to generate an input-output (I-O) curve. LTP was induced by applying two trains of standard theta-burst stimulation (TBS, each train containing 10 bursts of 5 pulses at 100 Hz delivered at 200 ms intervals, 30 s between each train) at baseline stimulus intensity. Baseline pulses were delivered every 30 s for 20 min prior to the TBS and for 60 min after. Baseline recording continued for a further 60 min. The initial slopes of the fEPSPs were measured, and each response expressed as a percentage change from baseline, defined as the average of the last 30 responses before TBS delivery. Initial LTP magnitude was calculated as the average slope of the first 20 responses after TBS, while the final LTP magnitude was calculated as the average slope of the last 20 responses, all normalised to the baseline.

#### Patch-clamp electrophysiology

Slice preparation was as for the field recording experiments except that animals were anaesthetised with pentobarbital (200 mg/kg; i.p.) and transverse whole hemisphere slices (400 μm) containing the dorsal hippocampus were cut. Hippocampal CA1 whole-cell recordings were performed in mice at 43–54 weeks of age. The Schaffer collateral pathway in stratum radiatum was stimulated using 50 μm Teflon-insulated tungsten monopolar electrodes and evoked excitatory postsynaptic currents (EPSCs) were recorded from visualised CA1 pyramidal cells using a glass patch electrode at holding voltages of -70 mV, -60 mV, -40 mV, -20 mV, 0 mV, + 20 mV and + 40 mV. Pipette resistance ranged from 4–5 MΩ and the internal solution consisted of the following: 145 mM CsMeSulfonate, 10 mM hydroxyethyl piperazineethanesulfonic acid (HEPES), 4 mM Na_2_ATP, 0.4 mM NaGTP, 5 mM QX-314, 4 mM MgCl_2_, 10 mM Na_2_phosphocreatine, 0.2 mM EGTA.4Na, 10 mM TEA, and 0.1 mM spermine dissolved in MilliQ water.

The stimulating electrode in stratum radiatum was placed 200–400 μm from the patch electrode in stratum pyramidale. Cells having resting membrane potentials of < 60 mV and exhibiting an input resistance of > 30 MΩ, and with an access resistance of 10–20 MΩ were included in the experiment. Cells were discarded if the access resistance changed by > 25% from their baseline values. Synaptic I/V plots were constructed by first, normalising each cell’s EPSC amplitude data as a percentage of the -60 mV response, and then plotting normalised average AMPAR synaptic current responses versus their respective holding potential from -70 to + 40 mV for each group. The Rectification Index (RI) was calculated from the synaptic I/V values as the ratio of EPSC amplitudes between -60 and + 40 mV. 1-Naphthylacetylspermine (NASPM) was used to selectively inhibit calcium-permeable AMPARs.

All EPSCs were recorded in voltage-clamp mode using a MultiClamp 700B (Molecular Devices) microelectrode amplifier and stimulation by the tungsten electrode was controlled through a custom-built programmable constant-current stimulator. Slices were imaged using differential interference contrast fitted to an Olympus BX50 upright microscope under a 40X objective. The following drugs were added to the aCSF: SR95531 (5 μM), gabazine (5 μM) and NASPM trihydrochloride (20 μM), all from Abcam; LY341495 (3 μM, cat# 1209), nimodipine (10 μM, cat# 0600), D-AP5 (100 μM, cat# 0106) and CGP55845 hydrochloride (1 μM, cat# 1248), all from Tocris.

#### Data analysis

pCLAMP version 10 (Molecular Devices) software was used for AMPAR-mediated EPSC data acquisition and analysis. Statistical significance was determined by univariate ANOVA followed by Bonferroni post hoc analysis for the different genotypes using Prism 9.0 software (Graph Pad Software). Data are reported as mean ± SEM.

### ECoG measurements

Prior to surgery, mice were housed in groups of up to four animals. Mice that underwent ECoG electrode implantation surgery and recordings were housed individually during recovery and for the duration of recordings.

#### Electro-corticogram (ECoG) electrode implantation surgery

Mice were surgically implanted at 40 weeks of age or older and allowed to recover for at least 7 days before recording. Isoflurane (IsoFlo; Abbott Laboratories) at a concentration of 4–5% mixed in O_2_ (vol/vol) was used for induction of anesthesia, maintenance was achieved with a 1–2% concentration. The depth of anesthesia was assessed by the absence of foot and tail pinch withdrawal reflex. A subcutaneous injection of meloxicam (1 mg/kg) dissolved in 0.9% saline was given prior to surgery. The mouse was placed in a stereotaxic frame (myNeuroLab, Leica Microsystems) and the scalp was shaved and sterilized with 80% ethanol. The skin was infiltrated with Lignocaine (1% ampules, Pfizer) and a 1 cm long incision was made. The skull was cleaned with 3% hydrogen peroxide solution (Pfizer) and four burr holes were drilled onto the skull. Epidural stainless-steel wire electrodes (Plastics One Inc., part no. MS 333-3A 0.005 inches) were implanted on the right frontal (reference electrode), left parietal (recording electrode) and occipital bone (ground electrode). An anchoring 1 mm screw was implanted in the occipital bone (extradural) for stability. The electrode pedestal and anchoring screw were embedded in methyl methacrylate dental cement (Catalog #1255710; Henry Schein Inc). Mice recovered in a Thermacage (Datesand Ltd) at 30 °C until fully awake.

#### Video-electrocorticographic recordings

Recordings were done via a tripolar cable (Plastics One Inc., Catalog# 335-340-3 0-SPR 80CM tripolar) and a commutator (Plastics One Inc., Catalog #8BSL3CXCOMMT) connected to a Grael EEG amplifier (Compumedics). The cables were connected to the head pedestal under light anesthesia (1–3% isoflurane for less than 5 min). Signals were band-pass filtered at 1 to 70 Hz and sampled at 256 Hz using Profusion data acquisition system (Compumedics) with simultaneous synchronized video monitoring (Profusion EEG4; Compumedics). Video recording was done using the Vivotek video server (VS8102) connected to an infrared day and night digital color camera (EVO2; Pacific Communications).

Mice were recorded for up to 5 days with the first 12 h of recording excluded. All ECoG traces were visually examined offline and events were manually counted. Spikes were defined as isolated events of an amplitude of equal or > 500 μV and a duration of up to 80 ms. The data was reported as the total number of spikes examined over a 24 h window. The presence of seizure activity was confirmed in video recordings.

### KA-induced seizures

Mice were 22 weeks of age (± 2 weeks) at the time of testing. Animals were administered kainic acid (KA; 25 mg/kg i.p.; Sigma-Aldrich Pty Ltd) and observed for 2 h. Mice were scored for seizure level based on the Racine scale ranging from 0–7 as per our previous method [58, 69].

### Cobalt uptake

Cobalt uptake experiments in mice (42 weeks of age ± 2 weeks) were conducted as previously described [58, 70].

### Immunohistochemistry

Mice (42 weeks of age ± 2 weeks) were anesthetised with a cocktail of ketamine (8.7 mg/mL) and xylazine (2 mg/mL) via i.p. injection, and transcardially perfused with 4% PFA. Brains were coronally sliced at 40 μm thickness using a cryostat (Leica Microsystems CM3050 S) and immunohistochemistry on free-floating sections were conducted as previously described [58, 65]. Sections were incubated in the following primary antibodies: mouse anti-NeuN (1:500; Millipore Cat# MAB377, RRID:AB_2298772, Merck Millipore), rat anti-CD68 (1:100; Bio-Rad Cat# MCA1957, RRID:AB_322219, Bio-Rad Laboratories), rabbit anti-GFAP (1:300; Agilent Cat# GA524, RRID:AB_2811722, Agilent Dako) and human anti-6E10 (1:1000; Covance Cat# SIG-39345-200, RRID:AB_662802, BioLegend). Following primary antibody incubation, tissue sections were subsequently incubated in the appropriate biotinylated secondary antibodies (1:250; Thermofisher Scientific).

### Stereology

Quantification of cell population estimates were made using a brightfield microscope (Zeiss Axio Imager A1) as previously described [58, 65]. All stereological cell counts were performed blind to genotype and age.

### Golgi impregnation

Under isoflurane gas anaesthesia, mice (42 weeks of age ± 2 weeks) were euthanized by cervical dislocation and brains were immediately removed. The brains were coronally sliced at 100 μm thickness using a cryostat (Leica Microsystems CM3050 S) and stained using the FD Rapid GolgiStain™ kit (FD NeuroTechnologies Inc, MD) as per the manufacturers’ recommendations. Sections were coverslipped with Permount™ (ThermoFisher Scientific) and allowed to dry for 24 h prior to analysis using a brightfield microscope (Zeiss Axio Imager A1). Dendritic morphology was assessed as previously described [58]. For each brain, 5 neurons from the hippocampal CA1 pyramidal layer were traced. CA1 spine density was assessed by counting the number of spines in 3 branches per neuron (5 neurons/brain) of branch orders 2–4 as previously described [58]. All protrusions < 2 µm were counted as spines provided they were continuous with the dendritic shaft. The spine density was defined as the number of spines per 10 µm of dendritic length.

### Amyloid assessment

#### 6E10 quantification

Quantification of the 6E10 staining was performed using the Image-Pro Plus v.6.0 image analysis system to analyze the percent area occupied by positive staining, as previously described [65].

#### Amyloid-β plaque quantification

Thioflavin S staining was used to determine the number of fibrillar Aβ plaques, as previously described [65]. WT mice were not assessed due to no detectable Thioflavin S being observed [71].

#### Amyloid-β ELISAs

Mice were cervically dislocated under isoflurane gas anaesthesia and the hippocampus was rapidly dissected, weighed and homogenised in 5 vol/wt of TBS (Tris-HCL 50 mM pH 7.6; NaCl 150 mM; EDTA 2 mM) containing a cocktail of protease inhibitors (1:100, Sigma-Aldrich Pty Ltd). Samples were then suspended in 2% SDS containing protease inhibitors (1:100, Sigma-Aldrich Pty Ltd) and centrifuged at 100,000 × *g* for 1 h at 4 °C. The supernatant containing the soluble Aβ fraction was collected. The remaining pellet was resuspended in 20 μl of 70% formic acid, homogenised and centrifuged at 100,000 × *g* for 1 h. Following centrifugation, 180 μL of Tris–HCL (1 M, pH 11) was added to neutralise the sample. The supernatant containing the insoluble Aβ fraction was collected. The Aβ levels were determined by using the BetaMark™ Total Beta-Amyloid Chemiluminescent ELISA Kit (Cat #: Covance Cat# SIG-38966-kit, RRID:AB_10718506; BioLegend), BetaMark™ Beta-Amyloid x-40 Chemiluminescent ELISA Kit (Cat #: SIG-38950; BioLegend) and BetaMark™ Beta-Amyloid x-42 Chemiluminescent ELISA Kits (Cat #: SIG-38952; BioLegend), as per manufacturers’ instructions. Previous studies have shown mice overexpressing hAPP, including J20 mice, exhibit a much larger signal in Aβ levels compared to WT mice [71, 72]. As such, only comparisons between J20 and Gria2^G/G^/J20 mice were conducted.

### Cytokine ELISAs

Quantification of the inflammatory cytokine TNF-α was conducted via antibody-specific ELISA. Mice were anesthetised with isoflurane, cervically dislocated, the hippocampus was removed, snap frozen and stored at -80 °C until use. The tissue was homogenised in 50 mM Tris-HCl, pH 7.2, 50 mM NaCl, 1% Triton X-100 and 50 mM Sodium Fluoride (NaF) containing protease inhibitors (1:1000, Sigma-Aldrich Pty Ltd). Samples were centrifuged at 14,000 × *g* for 10 min at 4 °C. The supernatant was collected and total protein concentration was determined using the Bradford Assay. TNF-α (BioLegend Cat# 430901, RRID:AB_2883995) protein concentrations were quantified by ELISA kit in accordance with the manufacturer’s instructions.

### Western blots

Mice were anesthetised with isoflurane, cervically dislocated and the hippocampus was rapidly dissected and frozen at -80 °C until use. Unless otherwise specified, tissue was homogenised by sonication in 500 μL RIPA buffer (Sigma-Aldrich Pty Ltd) and supplemented with a protease inhibitor cocktail (1:100, Sigma-Aldrich Pty Ltd). Proteins were resolved by SDS-PAGE on 4–12% Bis–tris gels (NW04122BOX, Thermofisher Scientific) in 1 × MES SDS running buffer (B0002, Thermofisher Scientific), transferred to polyvinylidene difluoride (PVDF) membranes (IB24001, Thermofisher Scientific) and blocked with 5% non-fat dry milk for 1 h at room temperature. Membranes were immunoblotted with primary antibodies overnight at 4 °C with agitation followed by a 1 h incubation with the appropriate horseradish-peroxidase (HRP)-conjugated secondary antibody at room temperature with agitation. Signals were developed with chemiluminescence (WP20005, Thermofisher Scientific) and exposed to film. Where appropriate, antibodies were removed with stripping buffer (100 mM 2-mercaptoethanol, 2% SDS, 62.5 mM Tris-HCl, pH 6.7) at 50 °C for 45 min, followed by washing for 1 h with tap water and re-probing membranes for β-tubulin. The intensity of bands was measured by using ImageJ software. The following antibodies were utilized: ADAR2 (1:1000, Santa Cruz Biotechnology Cat# sc-33180, RRID:AB_2222780), GluA1 (1:1000, Millipore Cat# AB1504, RRID:AB_2113602, Merck Millipore), GluA2 (1:1000, Abcam Cat# ab20673, RRID:AB_2232655, Abcam), GluA2/3 (1:1000, Millipore Cat# 07-598, RRID:AB_310741, Merck Millipore), GluA3 (1:1000, Cell Signaling Technology Cat# 3437, RRID:AB_1264115, Cell Signaling Technologies), β-tubulin (1:1000, Promega Cat# G7121, RRID:AB_430874, Promega), HRP-conjugated anti-rabbit IgG (1:5000, Millipore Cat# AP132P, RRID:AB_90264), and HRP-conjugated anti-mouse IgG (1:5000, Millipore Cat# AP124P, RRID:AB_90456).

### Co-immunoprecipitation

Co-immunoprecipitation experiments were conducted as previously described [58, 73]. Mice were anesthetised with isoflurane, cervically dislocated and the hippocampus was isolated and frozen at -80 °C until use.

### BS^3^ crosslinking

Bis(sulfosuccinymidal)suberate (BS^3^) is a cell-impermeable crosslinker which covalently bonds surface proteins to other nearby surface proteins thereby increasing their molecular weight. Specific crosslinked protein aggregates can then be identified via immunoblot detection. BS^3^ crosslinking therefore enables the discrimination of AMPARs located on the cell surface from intracellular AMPARs. BS^3^ crosslinking was performed as previously described [74]. Briefly, mice were anesthetized with isoflurane, cervically dislocated, the brain was rapidly removed, and the tissue was immediately mounted onto a vibratome. 400 μm coronal sections were taken from AP positions between bregma -1.34 mm and -2.3 mm. The hippocampus was then isolated from the slice, under a dissecting microscope. Hippocampal sections were added to 100 μL of ice-cold artificial cerebral spinal fluid (ACSF) that was immediately spiked with 2 mM of bis(sulfosuccinymidal)suberate (BS^3^; Thermofisher Scientific). For experiments conducted on CA1, CA3 and DG hippocampal regions, the regions were isolated under a dissecting microscope and incubated in ACSF with BS^3^ as above. Samples were incubated with agitation at 4 °C for 30 min, prior to the addition of 100 mM glycine for 10 min at 4 °C to terminate the reaction. Following crosslinking, tissue was centrifuged for 2 min at 17,000 × *g* to pellet the sample. The supernatant was discarded, and the pellet was resuspended in 200 μL of RIPA buffer (Sigma-Aldrich Pty Ltd) containing protease inhibitors (Sigma-Aldrich Pty Ltd) and homogenised by sonication. Samples were centrifuged at 17,000 × *g.* Samples were subjected to SDS PAGE and immunoblotting for GluA2 (Abcam Cat# ab20673, RRID:AB_2232655, Abcam).

### Capillary electrophoresis and immunoprobing

These methods pertain only to supplemental Fig. 5b as this data was acquired post relocating to a new laboratory. Tissue was collected from 5xFAD WT and transgenic littermates at 40 weeks of age (± 2 weeks) by anesthetising mice with 5% isoflurane in air and transcardially perfusing with PBS. Brain tissue was hemisected and snap frozen before being dissected to isolate the hippocampus and suspending in 500 μl RIPA buffer containing protease inhibitor cocktail (Sigma-Aldrich Pty Ltd Cat#S8830) and PhosSTOP™ phosphatase inhibitors (Sigma-Aldrich Pty Ltd Cat#4906845001). Hippocampal tissue was then homogenised by low amplitude sonication for 20 s and incubated on ice for 45 min. Resulting cell suspension was centrifuged at 14,000 rpm for 15 min at 4 ℃ and the supernatant isolated and stored at -80 ℃. The optimal antibody concentration, and linear dynamic ranges for ADAR2 was determined prior to conducting expression analysis.

To quantify ADAR2 protein expression, samples were run on a standard 25-well WES operating plate, as per manufacturer’s instructions using the WES Simple Western instrument (ProteinSimple). Reagents were obtained from 12-230 kDa separation modules (ProteinSimple Cat#SM-W004) and Total protein detection modules (ProteinSimple Cat#DM-TP01). A protein concentration of 0.3 μg/μl was used for both wildtype and mutant hippocampal tissue. A working dilution of 1:30 was used for the ADAR2 antibody (Santa Cruz Cat#SC33180, RRID: AB_2222780, Santa Cruz Biotechnology) in all experiments performed.

For detection of ADAR2 expression, samples were replicated in two separate wells within individual plates and treated with either anti-ADAR2 primary antibody or the total protein assay. The total protein assay functions in a similar way to a Coomassie-stained gel whereby a biotin is attached to all proteins in the sample and incubation with streptavidin-HRP followed by luminol and peroxide generates a chemiluminescent signal for total captured protein. An internal control sample (derived from wildtype mice) was run in technical duplicates for both the primary antibody and total protein concentration, so that data could be standardised to this internal calibrator across different plates. Within each plate, several wells were used to control for variables including background biotinylation for sample diluent in the absence of sample, background biotinylation for the sample in the absence of the biotinylation label and background antibody signal for the sample diluent in the absence of sample.

Data was analysed using the WES instrument software (ProteinSimple, Compass for SW 4.1 Windows 7/8/10 64 bit). Peak analysis settings were performed on the electropherograms (EPG) as follows: Range: (1–250); Baseline: threshold (0.1), window (400), stiffness (0.1); Peak Find: threshold (10), width (9), area calculation (Dropped lines). Baseline adjustments were made to fit relative background chemiluminescence signals with all samples measured at identical conditions. Dropped line analysis was preferred over a gaussian fit model to adjust for interfering additional peaks and for better control of relative peak signal. The ADAR2 antibody peak was identified approximately between 92–98 kDa. The resulting signal from the total protein assay yielded a broad multi-peak EPG and the cumulative area under these peaks was measured as the expression of all protein in the sample against which the target protein was normalised.

### Behavioural testing

Each behavioural testing trial included animals from all four genotypes. Behavioural testing was conducted using three different paradigms (one test per day) with separate cohorts of mice beginning at 24 weeks of age. Paradigm 1 consisted of open field test (OFT) followed by object recognition, elevated plus maze (EPM), Y-maze and working memory radial arm maze (RAM). Paradigm 2 consisted of OFT, object recognition, EPM, Y-maze and rotarod. Paradigm 3 consisted of reference memory RAM. After completion of testing, animals were euthanized for tissue collection studies at pre-determined ages.

#### Open field test

The OFT was performed as previously described [58, 65]. Briefly, the arena (40 × 40 cm) was situated in a large sound-attenuating box and had clear plexiglass walls, no ceiling, and a white floor (Med Associates Inc). The total distance traveled over 10 min was recorded. The arena was thoroughly cleaned with 70% ethanol (EtOH) between each mouse.

#### Elevated plus maze

The EPM was performed as previously described [65]. Briefly, the EPM consisted of four arms (77 × 10 cm) elevated (70 cm) above the floor (Med Associates Inc), two of which had 20 cm high walls (i.e. the ‘closed’ arms) and the remaining two had no walls (i.e. the ‘open’ arms). A video camera recorded the mouse and a computer software program (Limelight; Med Associates Inc) was used to measure the number of open arm entries and time spent in the open arms, as an indication of anxiety-like behavior. The ratio of open arm entries to total entries was analyzed.

#### Rotarod

Mice were place on a suspended rotating beam (Med Associates Inc) and the total time spent on the beam was recorded over three trials (1 trial per day for 3 days), as previously described [58].

#### Object recognition

The testing arena consisted of opaque plexiglass (50 × 30 cm) that was rectangular in shape, with 35 cm high walls. Two identical objects (red wooden blocks 6 × 4 × 3 cm in shape) were placed symmetrically 15 cm apart from each other and approximately 5 cm away from the arena walls. The protocol was similar to Heneka et al. [75], with slight variations; a single testing session, consisting of two trials 4 h apart, was conducted. During trial 1, the mouse was allowed to freely explore the identical objects for 10 min. During trial 2, the mouse was allowed to freely explore the arena for 5 min, however, this time, one object was replaced with the novel object (a wooden yellow arch 8 × 5 × 3 cm in shape). The arena and the objects were thoroughly cleaned with 70% EtOH between each mouse. All trials were video-recorded and the time spent exploring each object during each trial was recorded manually. Exploration was defined as directing the nose to the object at a distance of no more than 1 cm and/or touching the object with the nose. Data is presented as the discrimination ratio: time spent exploring the novel object/time exploring both objects.

#### Y-maze

The Y-maze was performed as previously described [75], with modification. Testing was conducted in an opaque plexiglass Y-shaped maze consisting of three arms (40 × 4 × 17 cm) diverging at a 120° angle (Med Associates Inc). Each mouse was placed in the centre of the Y-maze and allowed to explore freely through the maze during a video-recorded 5 min session. The sequence and total number of arms entered was recorded manually. Arm entry was counted when the hind paws of the mouse had been completely placed in the arm. Percentage alternation was calculated as the number of triads containing entries into all three arms divided by the maximum possible alternations (the total number of arms entered minus 2) × 100. The maze was thoroughly cleaned between each mouse with 70% EtOH.

#### Radial arm maze

The RAM consisted of eight arms (65 × 9 cm), extending radially from a central arena (35 cm diameter) and placed on a table elevated (90 cm) above the ground (Med Associates Inc). Each arm and the central arena were made of plexiglass, with enclosing walls made of clear plexiglass. Extra-maze cues consisted of the investigator, who was located in the same position for all trials, as well as large, fixed furniture around the room. The RAM was thoroughly cleaned with 70% EtOH between each mouse. Each food reward container was wiped with a small amount of sweetened condensed milk prior to the commencement of each trial to avoid the presence of olfactory cues. Additionally, the maze was rotated 45° after all mice had completed the trials for the day to avoid the use of intra-maze cues during training.

##### Working memory RAM

Mice were individually housed and restricted to 85% of their original body weight for 1 week prior to the commencement of RAM testing. On the first, second and third day, mice were habituated to the maze by being placed into the central arena, with one arm open and baited with sweetened condensed milk. Starting on the fourth day and continuing once a day for 12 days, mice underwent a working memory task, where all eight of the arms were baited with sweetened condensed milk. The training trial continued until all eight baits were retrieved or until 8 min had elapsed. Following testing, the mice were returned to their home cage. An investigator recorded the total number of entries into the arms and an error was marked when a mouse re-entered an already retrieved arm within the same trial. Data are presented as “Session”, consisting of 2 days (a total of two trials).

##### Reference memory RAM

Mice were individually housed and diet restricted to 90% of their original body weight for 1 week prior to the commencement of RAM testing. Reference memory RAM was performed twice daily for 24 days, as previously described [65]. An investigator recorded the number of successful entries into the baited arms (where the sweetened condensed milk was consumed) divided by the total number of entries made. Data are presented as “Session”, consisting of 2 days (a total of four trials).

### Statistical analysis

All statistical analyses were performed using the statistical package Prism 9 (GraphPad). For normally distributed data, differences between means were assessed, as appropriate, by one- or two- way ANOVA with or without repeated measures, followed by Bonferroni post hoc analysis. For non-parametric data, Kruskal-Wallis ANOVA was used, followed by Wilcoxon matched pairs signed-rank test. To assess differences between two groups, a student* t*-test was used. For t-tests, data sets were first tested for normality, before using parametric or non-parametric tests. For parametric tests, an F test for variance was used to determine whether standard deviations were equal between groups. If unequal, Welch’s correction was applied, as indicated. For non-parametric tests, the Mann-Whitney test was conducted, as indicated. All data is presented as mean ± SEM for line graphs, or mean ± SD for all other graphs, as indicated. For all statistical tests, a *p* value of ≤ 0.05 was assumed to be significant.

## Results

Extensive evidence indicates that GluA2 Q/R site editing is deficient in various regions of the human AD brain [21, 50–52]. We aimed to determine if preventing the expression of unedited GluA2(Q), and therefore only allowing the expression of GluA2(R), could prevent AD-related pathologies and cognitive phenotypes in the J20 mouse model of AD. To achieve this, we created a genetically-modified mouse encoding a CGG in place of a CAG in the *Gria2* Q/R editing site (Sup Fig. 1a)*.* An analogous nucleotide substitution (i.e. adenosine to inosine editing) is normally made by the ADAR2 enzyme in the pre-mRNA, post-transcription. Thus, by exonically encoding the edited arginine (R) amino acid, the need for editing to occur at this site is negated and GluA2(R) is expressed wherever GluA2 is normally expressed. DNA sequencing confirmed a guanine (G) nucleotide in the position that would otherwise harbor an adenosine (A) nucleotide in WT *Gria2* alleles (Sup Fig. 1b). Heterozygous and homozygous mice were identified by PCR of the downstream intronic loxP sequence (Sup Fig. 1c). We further verified homozygous mice did not express GluA2(Q) by performing RT-PCR of *Gria2* containing the Q/R editing site and subsequent digestion with *BbvI* restriction enzyme (Sup Fig. 1d).

To determine the effects of abolishing any possible unedited GluA2(Q) translation in a model of AD, we crossbred homozygous *Gria2*^tm1BViss^ mice with J20 mice. We assessed WT, Gria2^G/G^, J20 and Gria2^G/G^/J20 mice (see Table 1 for description of genotypes) across a battery of anatomical, behavioural and electrophysiological parameters. Gria2^G/G^ mice were viable and had no significant difference in body weight compared with WT littermates. In comparison to both WT and Gria2^G/G^ mice, both J20 and Gria2^G/G^/J20 mice had significantly reduced body weight at 24 weeks of age (Sup Fig. 2).

### Increased presence of Ca^2+^-permeable AMPARs in J20 mice is prevented in Gria2^G/G^/J20 mice

**Fig. 1:**
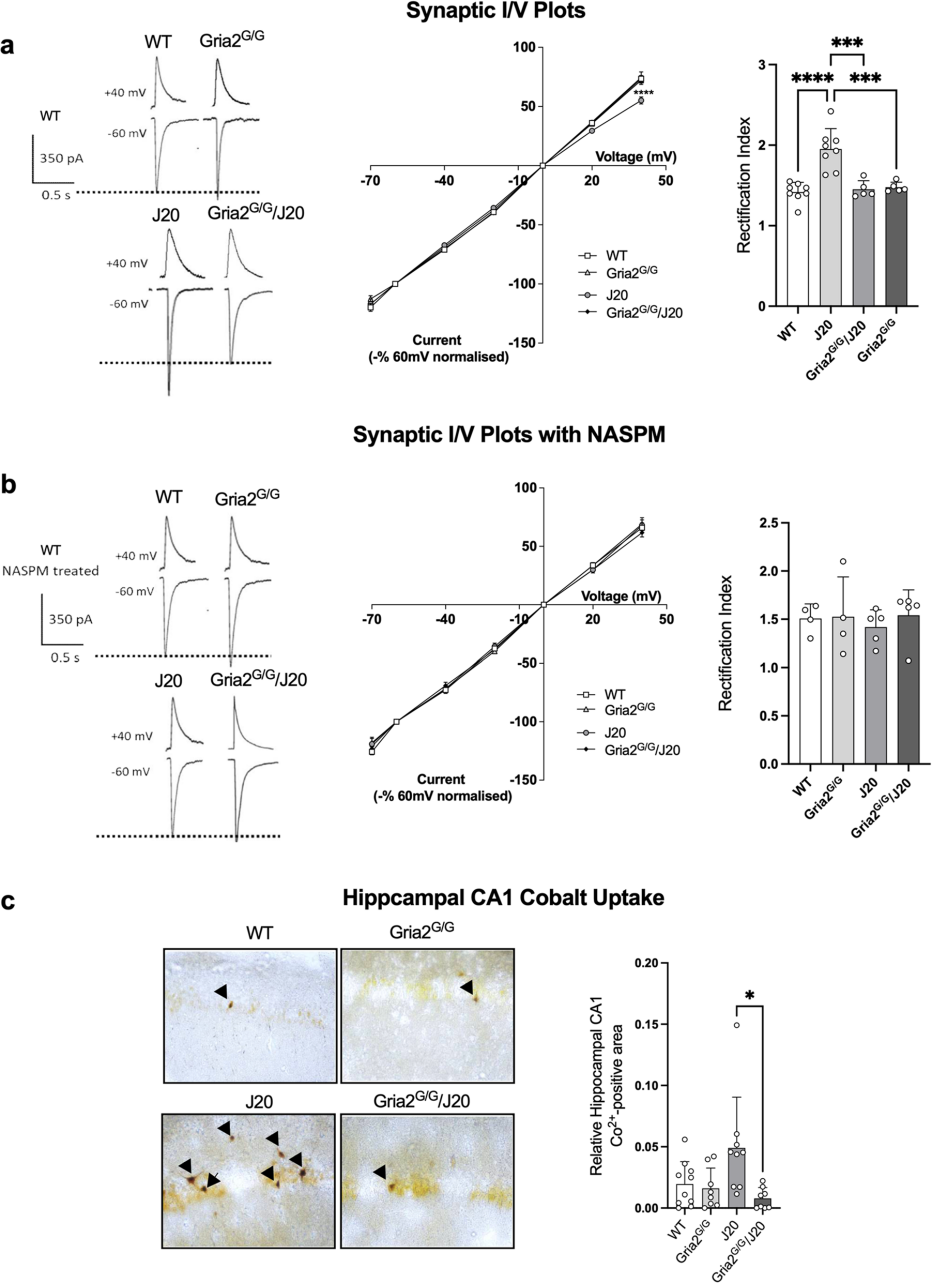
J20 mice exhibit Ca^2+^-permeable AMPA receptors. **a** Current-voltage (I/V) relationship of synaptic responses at holding potentials of -70, -40, -20, 0, +20 and +40 mV in WT, GluA2^G/G^, J20 and GluA2^G/G^/J20 mice normalized to evoked EPSC amplitude at -60 mV revealed inward rectification in J20 mice (*n’s*; WT = 8, GluA2^G/G^ = 5, J20 = 8, GluA2^G/G^/J20 = 5). The rectification index (RI), ratio of EPSC amplitudes at -60 mV and +40 mV, also confirmed a significantly larger inward current in J20 animals compared to all other genotypes. The representative waveforms for the WT group are absolute amplitude as per the scale bar. Representative waveforms for all other groups at both holding potentials are scaled equally to equate the +40 mV response with the WT +40 mV response. The dashed line indicates the peak amplitude of the WT -60 mV response. **b** The addition of NASPM, a Ca^2+^-permeable AMPAR antagonist, evoked linear I-V relationships in all four genotypes (*n’s*; WT = 4, GluA2^G/G^ = 4, J20 = 5, GluA2^G/G^/J20 = 5). Waveforms are as for panel **a**. **c** Representative Co^2+^ labelling images of the CA1 hippocampal region from WT, GluA2^G/G^, J20 and GluA2^G/G^/J20 mice. Kainate-induced Co^2+^ loading revealed a significant increase in the uptake of CoCl^2^ in acute slices taken from J20 mice, indicative of an increase in Ca^2+^-permeable AMPARs (*n’s*; WT = 10, GluA2^G/G^ = 8, J20 = 9, GluA2^G/G^/J20 = 8). Each value represents the mean ± the SD for bar graphs and SEM for line graphs. *p < 0.05, ***p < 0.001, ****p < 0.0001

A signature of Ca^2+^-permeable AMPARs is inwardly rectifying I/V relations [55]. We therefore assessed I/V relations and corresponding rectification index (RI) in all four genotypes to determine if this phenotype is present in J20 mice and, if so, if it is caused by the expression of unedited GluA2(Q)-containing AMPARs. We observed inwardly rectifying currents in hippocampal neurons of J20 mice (RI ANOVA; *F*_(3,22)_ = 17.38 *p* < 0.0001; Fig. 1a) but, notably, these were not present in Gria2^G/G^/J20 mice. In the presence of a specific inhibitor of Ca^2+^-permeable AMPARs (NASPM), channel conductance in J20 mice reverted to normal, illustrating the altered I/V relations in J20 mice are driven by the presence of Ca^2+^-permeable AMPARs (RI ANOVA; *F*_(3,14)_ = 0.20 *p* = 0.89; Fig. 1b).

The presence of Ca^2+^-permeable AMPARs was also demonstrated by the enhanced uptake of Co^2+^ in the CA1 region of the hippocampus in J20 mice. The uptake of CoCl^2^ by neurons is known to occur selectively via Ca^2+^-permeable AMPARs [70]. We found significantly increased Co^2+^ uptake in hippocampal CA1 neurons of J20 mice following stimulation with AMPA as compared to Gria2^G/G^/J20 mice, indicating increased Ca^2+^-permeable AMPARs in the J20 mice, but not in J20 mice where unedited GluA2(Q) expression was eliminated (ANOVA; *F*_(3,31)_ = 4.566 *p* = 0.009; Fig. 1c). The Co^2+^ uptake was blocked by the AMPA receptor antagonists GYKI and NBQX, demonstrating that the uptake was specific to Ca^2+^-permeable AMPARs (Sup Fig. 3). Given that both the altered I/V relations and the increased Co^2+^ labelling in J20 mice were prevented in mice with genetically encoded edited GluA2(R), these results suggest unedited GluA2(Q)-containing AMPARs are the primary cause of enhanced Ca^2+^ permeability in J20 mice.

Because our introduced mutation only affects GluA2-containing AMPARs and does not affect the presence of GluA2-lacking Ca^2+^-permeable AMPARs, we sought to confirm there were no changes in the expression of GluA2-lacking AMPARs in J20 mice. To do this, we assessed the surface and intracellular expression of GluA2 in whole hippocampal homogenates and in specific subregions of the hippocampus. We found no evidence of alterations in the surface or intracellular expression of GluA2 in the whole hippocampus (Sup Fig. 4a) or in sub-hippocampal regions (CA1, CA3 and DG; Sup Fig. 4b). Additionally, we found no evidence of alterations in the protein expression of total GluA2 or GluA2/3 in whole hippocampal homogenates (Sup Fig. 4c-d). Finally, we performed co-immunoprecipitations to determine if there were any changes in AMPAR composition, finding no significant differences between any of the four genotypes (Sup Fig. 4e). These results indicate there are no alterations to AMPAR complexes in J20 mice, and no changes in receptor architecture as a result of genetically encoding the edited GluA2(R) subunit in Gria2^G/G^ mice.

Given our results above strongly indicate enhanced Ca^2+^-permeability is a result of unedited GluA2(Q)-containing AMPARs in J20 mice, we also measured the hippocampal expression of the enzyme responsible for editing at this site, ADAR2. Consistent with literature from human AD, we found a significant reduction in ADAR2 protein expression in aged J20 mice (Sup Fig. 5a) as well as in aged 5xFAD mice (Sup Fig. 5b), another commonly used model of AD. Together, these results further corroborate our findings and suggest ADAR2 dysregulation may be causing a reduction in GluA2 Q/R site editing in J20 mice.

### Gria2^G/G^/J20 mice show prevention of CA1 hippocampal dendritic spine and neuron loss despite ongoing amyloid pathology

**Fig. 2:**
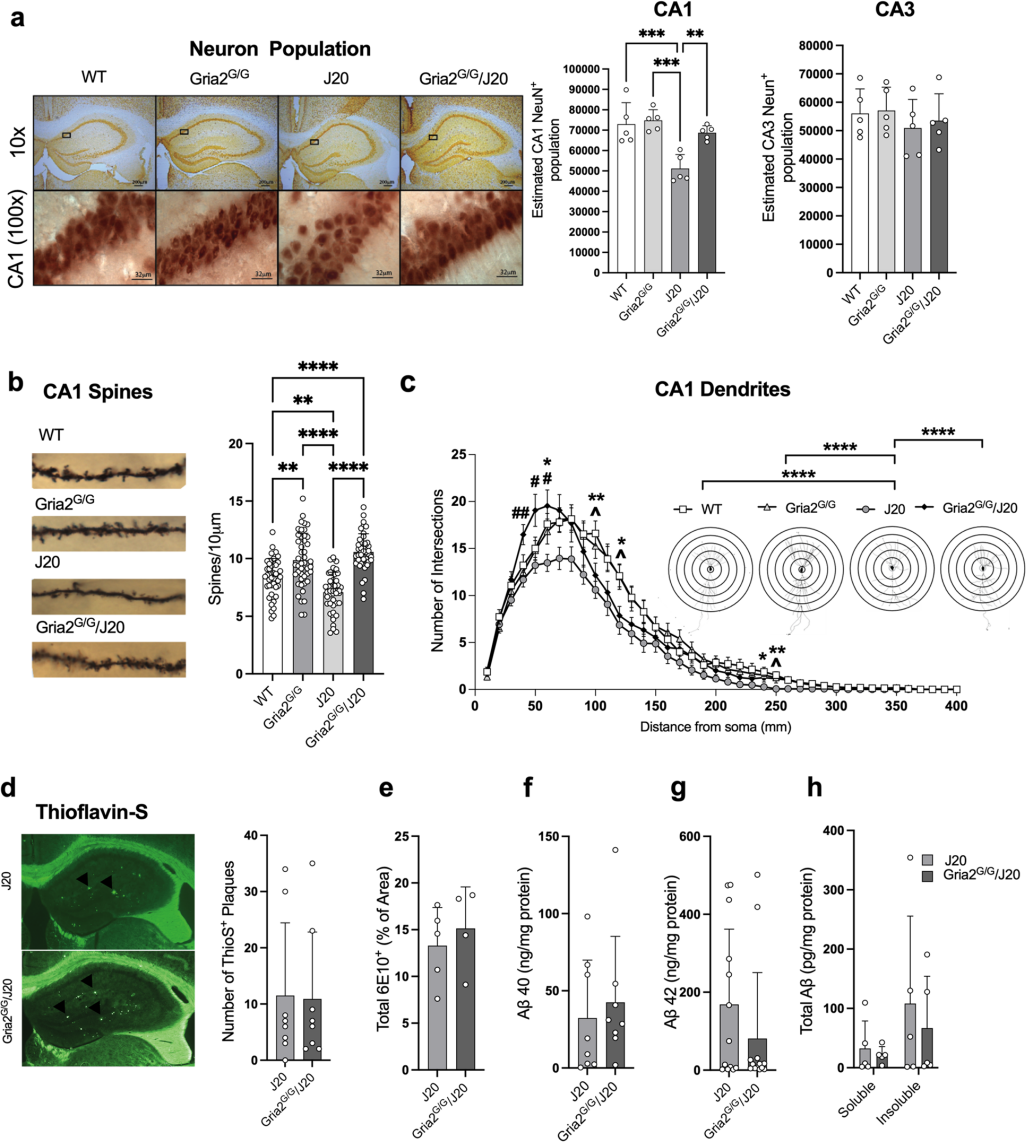
Genetically encoding GluA2(R) in J20 mice rescues neuronal and spine numbers but does not alter Aβ-pathology. **a** NeuN^+^ neurons in the hippocampus of WT, GluA2^G/G^, J20 and GluA2^G/G^/J20 mice. Stereological quantification of neuronal numbers revealed a significant decrease in the CA1 neuronal population of J20 mice compared to all other genotypes (*n* = 5/genotype) while no changes to CA3 neuronal populations were observed (*n* = 5/genotype). **b** Representative images of Golgi-stained CA1 dendritic spines in WT, GluA2^G/G^, J20 and GluA2^G/G^/J20 mice. Quantification of spine density revealed a significant reduction of spines in J20 mice compared to all other genotypes (*n* = 3 dendritic branches/neuron with 3 neurons/brain and 5 brains/genotype). Both GluA2^G/G^ and GluA2^G/G^/J20 mice displayed significantly higher spine density than WT littermates. **c** Scholl analysis and representative traces of Golgi-impregnated neurons from the hippocampal CA1 revealed significantly reduced numbers of dendritic intersections in J20 mice at varying distances from the soma compared to WT (*), GluA2^G/G^ (^) and GluA2^G/G^/J20 (#) animals (*n* = 3 neurons/brain and 5 brains/genotype). **d** Representative image of Thioflavin-S^+^ plaques in J20 and GluA2^G/G^/J20 mice. Quantification revealed no significant differences in Thioflavin-S^+^ plaques (*n* = 8/genotype). No differences in other Aβ species including (**e**) total 6E10^+^ area (*n’s*; J20 = 5, GluA2^G/G^/J20 = 4), (**f**) Aβ40 (*n* = 8/genotype), (**g**) Aβ42 (*n* = 13/genotype), or (**h**) total soluble and insoluble Aβ (*n* = 5/genotype) were observed between J20 and GluA2^G/G^/J20 mice. **a**-**b** Analysed using univariate ANOVA with Bonferroni correction, **c** analysed using two-way ANOVA with Bonferroni correction, **d**-**g** analysed using t-tests with Welch’s corrections and **h** analysed using either Mann-Whitney test (soluble fraction) or unpaired t-test (insoluble fraction). Each value represents the mean ± the SD for bar graphs and SEM for line graphs. **p < 0.01, ***p < 0.001, ****p < 0.0001

Synaptic and neuronal degeneration, particularly in the hippocampus, is a major pathological feature in the human AD brain [4–15, 18]. We have previously shown the J20 mouse model of AD shows age-dependent neurodegeneration in the CA1, but not CA3, region of the hippocampus [65]. In the present study, stereological quantification corroborated our previous result revealing significant CA1 hippocampal neuron loss in J20 mice at 44 weeks of age, compared to WT and Gria2^G/G^ mice (ANOVA; *F*_(3,16)_ = 11.89 *p* < 0.001; Fig. 2a – CA1), but no evidence of CA3 neuron loss (ANOVA; *F*_(3,16)_ = 0.46 *p* = 0.72; Fig. 2a – CA3). Remarkably, there were significantly more CA1 neurons in Gria2^G/G^/J20 compared to J20 mice, suggesting that genetically encoding edited GluA2(R) prevents age-related CA1 hippocampal neuron loss in J20 mice.

We next determined the effect of exonically encoding edited GluA2(R) on the spine density of second order dendritic branches of apical dendrites within the CA1 stratum radiatum of Golgi-impregnated tissue. Consistent with our neuronal analysis, we found a significant reduction of spine density in J20 mice at 44 weeks of age compared to WT and Gria2^G/G^ mice (Welch’s ANOVA; *W*_(3,96.11)_ = 36.52 *p* < 0.001; Fig. 2b). Post-hoc analysis revealed Gria2^G/G^/J20 mice had significantly more spine density than J20 mice (*p* < 0.001), indicating the elimination of unedited GluA2(Q) transcripts from J20 mice prevents spine loss pathology. Unexpectedly, we also observed that both Gria2^G/G^ (*p* < 0.01) and Gria2^G/G^/J20 (*p* < 0.001) mice had significantly more spine density than their WT littermates. This tantalisingly suggests a previously unidentified role for editing at the GluA2 Q/R site in regulating spine numbers in the hippocampus.

We next assessed the dendritic arborisation of CA1 neurons via Scholl analysis of Golgi-impregnated tissue. A significant interaction effect occurred, indicating an overall change in the number of intersections that were different between genotypes (Two-Way RM ANOVA *F*_(117,2184)_ = 2.691 *p* < 0.0001; Fig. 2c). Assessing the main effects revealed J20 mice had a significant deficit in the overall number of intersections 0–300 µm away from the cell soma compared with WT and Gria2^G/G^ mice. Post-hoc analysis revealed significant differences between WT and J20 mice (* symbols) as well as demonstrating the Gria2^G/G^/J20 mice had significantly more intersections 0–300 µm away from the cell soma compared to J20 animals (Fig. 2c, # symbols). Importantly, Gria2^G/G^/J20 mice did not differ in the number of dendritic branches from WT and Gria2^G/G^ littermates. These results therefore suggest unedited GluA2(Q) may impair dendritic complexity in the J20 mouse model of AD.

Despite finding significantly more neurons, spines and dendritic complexity in Gria2^G/G^/J20 mice compared to J20 mice, we found no gross reduction in the expression of AD-related pathologies including Thioflavin S^+^ amyloid plaques (*p* = 0.92; Fig. 2d), 6E10 staining of Aβ (*p* = 0.55; Fig. 2e), soluble Aβ40 (*p* = 0.62; Fig. 2f) and Aβ42 (*p* = 0.23; Fig. 2g) or total soluble (*p* = 0.69) or insoluble Aβ (*p* = 0.61; Fig. 2h). Furthermore, we found no significant changes in the total population of GFAP^+^ astrocytes (Sup Fig. 6a), CD68^+^ microglia (Sup Fig. 6b) or TNF-α levels (Sup Fig. 6c) between Gria2^G/G^/J20 and J20 mice. These results suggest the rescue of neuronal, synaptic and dendritic pathology in Gria2^G/G^/J20 mice is independent of alterations in Aβ pathology and neuroinflammation.

### Genetically encoding GluA2(R) at the Q/R site does not affect seizure susceptibility

**Fig. 3:**
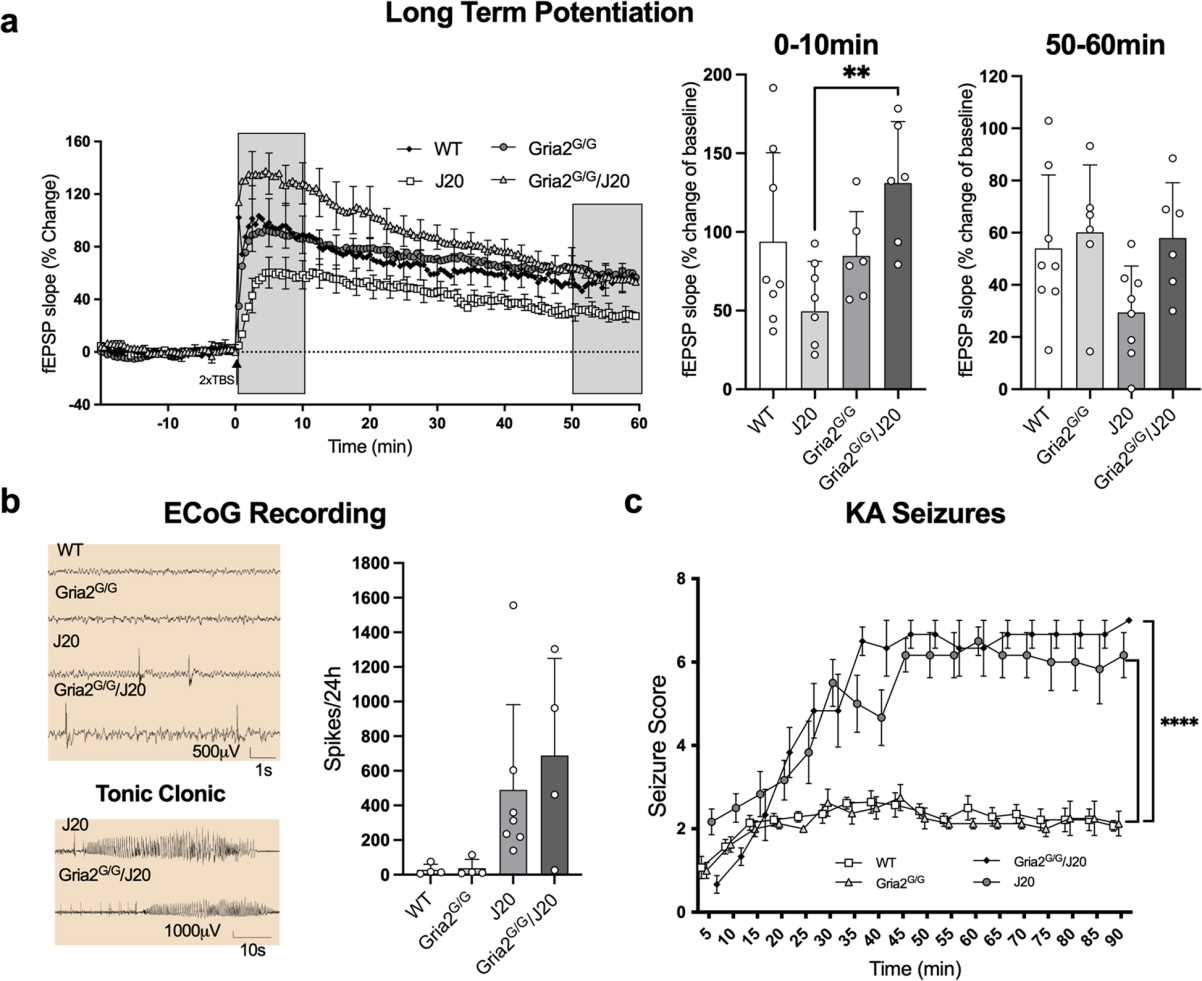
LTP and seizure phenotypes in J20 mice. **a** fEPSPs following 2 trains of TBS to the Schaffer collateral pathways evoked LTP in the hippocampus of all four genotypes (*n’s*; WT = 8, GluA2^G/G^ = 6, J20 = 8, GluA2^G/G^/J20 = 6). fEPSP slope analysis of 0–10 and 50–60 min post-TBS showed an increase in LTP induction in GluA2^G/G^/J20 mice compared to J20 mice (0–10 min) and a strong, but not significant, trend towards impaired LTP in J20 mice compared to all other genotypes (50–60 min), respectively. **b** Spike analysis over 24 h in genotypes showed a trend towards significance of J20 and GluA2^G/G^/J20 mice for increased seizure activity (*n’s*; WT = 4, GluA2^G/G^ = 4, J20 = 7, GluA2^G/G^/J20 = 4). Representative ECoG spike traces and tonic clonic seizure examples. **c** Kainic acid-induced seizure activity in J20 and GluA2^G/G^/J20 mice was significantly increased as compared to WT and GluA2^G/G^ mice (*n’s*; WT = 14, GluA2^G/G^ = 8, J20 = 6, GluA2^G/G^/J20 = 6). Each value represents the mean ± the SD for bar graphs and SEM for line graphs. **p < 0.01, ****p < 0.0001

Synaptic plasticity has long been considered one of the physiological mechanisms of learning and memory in the brain [76]. Thus, it is no surprise that mechanisms of synaptic plasticity, including long-term potentiation (LTP), appear to be disrupted in patients with AD [77] as well as in mouse models of AD [78]. Analysis of neurotransmission in the Schaffer collateral pathway of the CA1 hippocampal region showed a significant facilitation of the initial 10 min of LTP in the Gria2^G/G^/J20 mice compared to the J20 mice (ANOVA; *F*_(3,24)_ = 4.536 *p* < 0.01; Fig. 3a – 0–10 min). Though not statistically significant, there was also a trend towards a reduction in the final 10 min of LTP in J20 mice that was absent in Gria2^G/G^/J20 mice (ANOVA; *F*_(3,24)_ = 2.691 *p* = 0.069; Fig. 3a – 50–60 min). Additional investigations revealed no differences in basal synaptic transmission as assessed by input/output curves (Sup Fig. 7a) and no significant differences in paired-pulse facilitation over a range of inter-stimulus intervals (Sup Fig. 7b), indicating intact presynaptic mechanisms in the J20 mice and no effect of genetically encoding edited GluA2(R).

Spontaneous seizures are also a common, but less well characterised feature of AD [79, 80]. The J20 mouse model is unique in that it is one of the few mouse models of AD to display this phenotype [81]. Furthermore, mice that contain increased GluA2(Q) are seizure susceptible [35, 56–58], raising the hypothesis that GluA2(Q) expression may be causing the seizure phenotype in J20 mice. To determine if disturbances in unedited GluA2(Q) are implicated in seizure vulnerability in this model, we examined interictal discharges (“spikes”) from electrocorticography (ECoG) recordings over a 5-day period. As expected, WT and Gria2^G/G^ mice displayed no abnormal activity during the recording period. In contrast, numerous high-amplitude spikes, indicative of epileptiform activity, were detected in both J20 and Gria2^G/G^/J20 mice. Mean spike frequency averaged over a 24 h period showed a clear trend towards increased number of spikes in the J20 and Gria2^G/G^/J20 mice, though this was not statistically significant (ANOVA; *F*_(3,15)_ = 2.9 *p* = 0.07; Fig. 3b). Furthermore, at least one tonic-clonic convulsion, and one seizure detected only by ECoG, were observed in both J20 and Gria2^G/G^/J20 mice, indicating hyperexcitability in J20 mice, regardless of whether the edited GluA2(R) subunit was genetically encoded (Fig. 3b – Tonic Clonic).

To expand on these findings, we examined seizure vulnerability in response to a low i.p. dose of the excitotoxin, kainic acid (KA). An interaction effect occurred indicating a difference in seizure vulnerability over time between genotypes (mixed effects analysis *F*_(69,689)_ = 6.5 *p* < 0.001; Fig. 3c). KA induced mild seizure activity in WT and Gria2^G/G^ mice, and this was significantly enhanced in both J20 and Gria2^G/G^/J20 mice (Fig. 3c). J20 mice displayed high seizure activity that was not prevented in the Gria2^G/G^/J20 mice. This suggests spontaneous seizures and susceptibility to seizures are not related to unedited GluA2(Q) but are instead driven by other mechanisms. Taken together, these results suggest genetically encoding edited GluA2(R) may modulate the induction phase of LTP in J20 mice, but that edited GluA2(Q) is not responsible for the seizure phenotype of J20 mice.

### Edited GluA2(R) prevents working and reference memory deficits in J20 mice

**Fig. 4:**
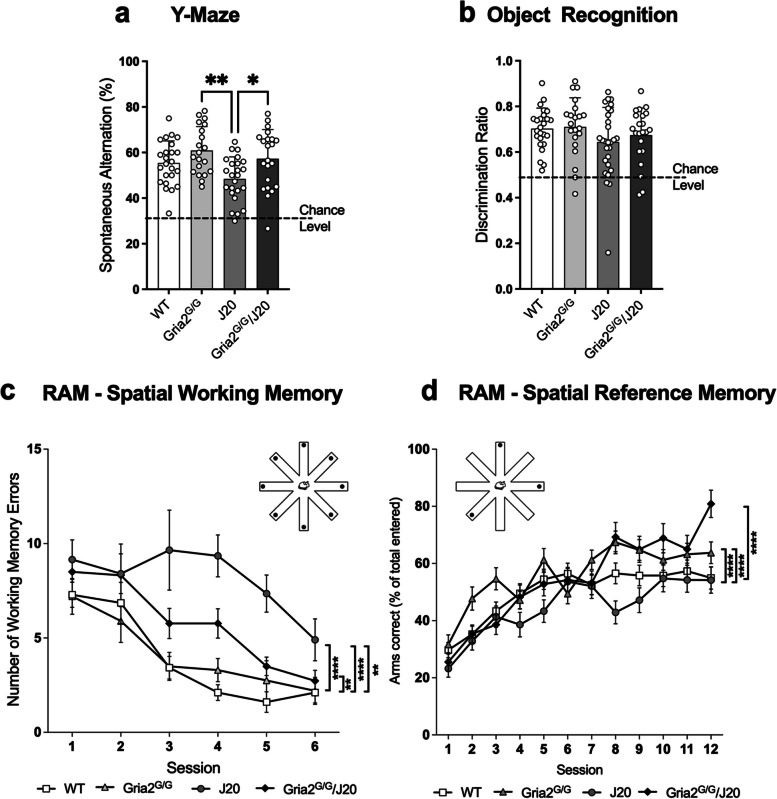
Genetically encoding GluA2(R) rescues various aspects of memory and learning in J20 mice. **a** GluA2^G/G^ and GluA2^G/G^/J20 mice showed a significant improvement in the percentage of correct alternations as compared to J20 mice in the Y maze (*n’s*; WT = 24, GluA2^G/G^ = 20, J20 = 24, GluA2^G/G^/J20 = 22). **b** No difference between all four genotypes in the discrimination between objects in the object recognition test (*n’s*; WT = 25, GluA2^G/G^ = 21, J20 = 28, GluA2^G/G^/J20 = 22). **c** In the working memory version of the radial arm maze, all eight arms were baited. J20 mice exhibited a significant increase in the number of errors compared to all other genotypes, that was partially recovered in GluA2^G/G^/J20 mice (*n’s*; WT = 14, GluA2^G/G^ = 10, J20 = 10, GluA2^G/G^/J20 = 11). **d** In the reference memory version of the radial arm maze, three of the eight arms were baited. J20 mice exhibited increased number of errors as compared to all other genotypes (*n’s*; WT = 17, GluA2^G/G^ = 10, J20 = 8, GluA2^G/G^/J20 = 12). Each value represents the mean ± the SD for bar graphs and SEM for line graphs *p < 0.05, **p < 0.01, ****p < 0.0001

We, and others, have previously described impairments to memory and cognition in the J20 mouse model across a battery of behavioural assessments [65, 80, 82]. To assess the effect of genetically encoding edited GluA2(R) on memory phenotypes in J20 mice, we tested mice in a Y-maze, object recognition and in two versions of the radial arm maze (RAM). In the Y-maze test, mice with intact spatial working memory tend to navigate the Y-shaped maze by visiting the least recently entered arm, which can be measured as spontaneous alternations [83]. We assessed short-term spatial working memory in the Y-maze by calculating the number of spontaneous alternations as a percentage of total arm entries. Spontaneous alternation behaviour was significantly different between groups (ANOVA *F*_(3,86)_ = 5.36 *p* < 0.01; Fig. 4a). Post-hoc analysis revealed alternations in J20 mice were significantly reduced compared to Gria2^G/G^ (*p* < 0.01) and Gria2^G/G^/J20 (*p* < 0.05) mice. There were no differences in the total number of arm entries between any of the groups confirming the reduction of alternations in J20 mice is a reflection of a spatial working memory deficit which is rescued in Gria2^G/G^/J20 mice (Sup Fig. 8a).

The novel object recognition test was also conducted to identify alterations in recognition memory as a measure of parahippocampal region-dependent cognition [84]. No alterations in recognition memory were observed in any of the genotypes as all mice displayed a discrimination ratio well above chance level (ANOVA *F*_(3,92)_ = 1.476 *p* = 0.23; Fig. 4b) and spent the same amount of time exploring the novel object (Sup Fig. 8b).

The RAM is a hippocampal-dependent spatial memory and learning task that can determine working and reference memory function [85, 86]. For the working memory version of the RAM, all eight arms of the maze were baited with a food reward and an error was marked when a mouse re-entered an arm from which it had already collected bait (Fig. 4c). A two-way ANOVA with repeated measures revealed a main effect of time (*F*_(3.8,154.9)_ = 18.39 *p* < 0.0001) and genotype (*F*_(3,41)_ = 21.25 *p* < 0.0001). J20 mice had significantly more errors across the 6 sessions compared with all other genotypes, including Gria2^G/G^/J20 mice (*p* < 0.01; Fig. 4c). However, Gria2^G/G^/J20 mice still had significantly more errors than WT mice (*p* < 0.01; Fig. 4c), indicating a partial return of working memory toward the WT level. There were no differences in the time taken to complete the task between any of the genotypes indicating no difference in sensorimotor function between genotypes, and that time was not a limiting factor (Sup Fig. 8c).

For the reference memory version of the RAM, three of the eight arms were baited with a food reward and an error was marked when a mouse entered non-baited arms or re-entered an arm that they had already collected bait from (Fig. 4d). We have previously described that the J20 mouse model of AD exhibits deficits in spatial memory and learning during disease progression in the reference memory version of the RAM [65]. A two-way ANOVA with repeated measures revealed a main effect of time (*F*_(10.25,1886)_ = 38.39 *p* < 0.0001) and genotype (*F*_(3,184)_ = 15.12 *p* < 0.0001). Here, we again found a significant decline in reference memory in J20 mice compared to WT (*p* < 0.0001), but also compared to Gria2^G/G^ (*p* < 0.0001) and Gria2^G/G^/J20 mice (*p* < 0.0001; Fig. 4d). Remarkably, Gria2^G/G^/J20 mice showed no differences from WT and Gria2^G/G^ mice indicating full restoration of spatial reference memory (Fig. 4d).

To identify changes in locomotive and emotional phenotypes, we administered a range of sensorimotor tests. We and others have previously shown that J20 mice exhibit increased locomotion in the open field test (OFT) [65], indicating a hyperactive phenotype. Here, this phenotype was again apparent, with J20 mice travelling significantly more than WT and Gria2^G/G^ mice (Sup Fig. 8d). There was no significant difference between J20 mice and Gria2^G/G^/J20 mice, suggesting no prevention of this phenotype by genetically encoding GluA2(R) (Sup Fig. 8d). In an elevated plus maze (EPM), J20 mice have previously shown a disinhibition-like phenotype [82]. Here, we found J20 mice have a strong trend toward increased open arm entries as a ratio of total arm entries in the EPM, similar to what we have previously observed in this model [65]. This trend was not apparent in Gria2^G/G^/J20 mice (Sup Fig. 8e). In a 3-day rotarod paradigm, despite J20 and Gria2^G/G^/J20 mice outperforming Gria2^G/G^ animals on the first testing day, all groups successfully increased their latency to fall over the 3-day testing period, which is indicative of normal motor learning (Sup Fig. 8f). Combined, our battery of memory and motor tests indicate that genetically encoding ‘edited’ GluA2(R) restores hippocampal-dependent spatial navigation to wild-type levels in the J20 mouse model of AD.

## Discussion

Spine and neuronal pathology are thought to be the major physiological basis of cognitive decline in AD [14–16]. Furthermore, calcium dyshomeostasis has long been implicated in AD pathogenesis (see “the calcium hypothesis”) [59]. By genetically encoding GluA2(R), which prevents Ca^2+^ flux through GluA2-containing AMPA receptors, this study provides the first evidence that RNA editing at the Q/R site of GluA2 may act as a switch that regulates dendritic spine numbers both in health and in AD. These phenotypic improvements occurred despite ongoing presence of other AD hallmarks. Our findings are consistent with the notion that dendritic spine plasticity is regulated by RNA editing and that dysregulation of this process leads to spine loss, neurodegeneration and cognitive dysfunction in a mouse model of AD.

### J20 mice exhibit Ca^2+^-permeable AMPA receptors

When edited at the Q/R site, GluA2 renders AMPARs impermeable to Ca^2+^. However, AMPARs that are GluA2 lacking, or that contain unedited GluA2(Q), are Ca^2+^-permeable and show an inwardly rectifying I/V relationship [28, 55, 69, 87, 88]. It is widely presumed the majority of AMPARs at excitatory synapses are GluA2(R)-containing, and therefore Ca^2+^-impermeable [89]. Thus, When Ca^2+^-permeable receptors are found in wild-type mice, such as those seen at glutamatergic synapses onto inhibitory interneurons, they are GluA2-lacking [90]. GluA2-lacking receptors are thought to play a role in synaptic plasticity and learning [69, 88], however, Ca^2+^-permeable receptors resulting from the presence of unedited GluA2(Q) are thought to occur primarily only in pathological circumstances [61].

Our work indicates that J20 mice have significant inward rectification of Schaffer collateral excitatory synaptic currents in CA1 pyramidal cells. This is indicative of either GluA2-lacking receptors or of receptors containing unedited GluA2(Q). Crucially, however, our data illustrates that inward rectification is abolished in Gria2^G/G^/J20 mice, suggesting unedited GluA2(Q)-containing AMPARs are the primary driver of inward rectification in J20 mice. This finding was further supported by the increased cobalt uptake in the CA1 region of J20 mice which was also prevented in Gria2^G/G^/J20 mice. Additionally, we found a significant reduction in J20 mice of ADAR2 expression, the enzyme responsible for mediating GluA2 Q/R site editing, suggesting reduced editing was likely the primary reason for the altered current rectification.

Previously, an enhanced presence of Ca^2+^-permeable AMPARs has been observed in the APP/PS1 model of AD [91] as well as in hippocampal neurons infused with oligomeric amyloid-β [92]. Our study corroborates these prior works and furthers them by implicating unedited GluA2(Q) as the key source of enhanced AMPAR Ca^2+^-permeability. Genetically encoding GluA2(R) in our mice did not change the overall expression of GluA2 nor the expression of AMPAR receptor subtypes (i.e. the presence of GluA2-lacking AMPARs remained unchanged in our mice; see Sup Fig. 4). Thus, expression of Ca^2+^-permeable AMPA receptors that are normally GluA2 lacking, such as those seen in inhibitory interneurons, was unchanged in our mice. Collectively, our findings suggest the expression of unedited GluA2(Q) is functionally implicated in synaptic signalling abnormalities in J20 mice.

### Abolishing GluA2(Q) expression in J20 mice rescues dendritic abnormalities as well as neuron loss in the CA1, independently of amyloid-β

In confirmation of our previous study [65] we observed a significant decrease in CA1, but not CA3, neuron numbers in J20 mice, compared with littermate controls. We also observed significantly reduced dendritic spine density and dendritic arborisation in J20 mice, compared with littermate controls [67, 93–98]. Remarkably, genetically encoding edited GluA2(R) led to full restoration of the CA1 neuronal population and significantly increased spine density as well as the complexity of dendritic architecture in the J20 mouse model. These observations suggest unedited GluA2(Q) may be a major mechanistic driver of dendritic abnormalities, spine loss and neuronal dysfunction in J20 mice. This hypothesis is supported by evidence from us and others showing that enhanced expression of unedited GluA2 (i.e. Ca^2+^-permeable receptors) causes synaptic and neuronal degeneration in the hippocampus [56–58, 99].

Interestingly, the phenotypic improvements we observed in Gria2^G/G^/J20 mice occurred without any alteration to Aβ-pathology. This could be interpreted in two ways:

1. The specific phenotypes for which we observed an improvement may be aetiologically independent of Aβ-pathology in J20 mice. It is possible, for instance, these phenotypes instead relate to detrimental effects of APP mutations and APP overexpression on full-length APP function, or the function of APP cleavage products other than Aβ. We have previously reviewed this question [17, 48]. This interpretation would suggest GluA2 Q/R site dysregulation may cause these phenotypes downstream of APP dysfunction.
2. Dysregulation of GluA2 Q/R site editing and incorporation of unedited GluA2(Q) into AMPARs may be triggered by Aβ-pathology and may therefore be a major mechanistic pathway through which Aβ-pathology causes these phenotypes. This interpretation would fit well with the hypothesis of an amyloid-independent phase of AD [100], a phase in which dysregulation of GluA2 Q/R site editing may play a major role in driving the development of some AD-phenotypes. Both possibilities are worth exploring in future work.

Irrespective of how the phenotypes are caused, our data raises the possibility that neurodegeneration and memory loss in mouse models of AD may be prevented by targeting the Q/R site of GluA2, even in the presence of continuing Aβ-pathology. This corroborates findings from other studies indicating some AD-related phenotypes can be altered in AD mouse models independently of effects on Aβ-pathology [68, 101–103], suggesting some phenotypes of AD in mouse models and in humans are preventable or recoverable without having to remove Aβ-pathology from the brain.

### J20 mice have memory deficits which are prevented through genetically encoding GluA2(R)

J20 mice have behavioural abnormalities as well as learning and memory deficits across a range of paradigms. We previously reported hyperactivity in an open field test, and spatial reference memory deficits in a radial arm maze, but no alterations in anxiety on an elevated plus maze, or in fear memory encoding in a context fear conditioning paradigm [65]. Others have reported hyperactivity in open field [80, 82, 104–106] and Y-maze [105], reduced anxiety (or disinhibition) in the elevated plus maze [82, 104–107], inhibited startle responses [107] and learning and memory deficits in multiple paradigms including the Morris water maze [80, 82, 95, 101, 104–112], Y-maze [113], radial arm water maze [114], cross maze [111], the cheeseboard task [115] and novel object recognition [82].

In the present study, genetically encoding edited GluA2(R) rescued the J20 phenotype by improving both spatial working memory and spatial reference memory in the RAM as well as in the Y-maze. The hippocampus, in concert with other brain regions, contributes to the formation and retrieval of spatial memories [116, 117]. We therefore propose the prevention of dendritic spine and neuron loss and the resulting preservation of hippocampal connectivity and plasticity in the CA1 region of the hippocampus of Gria2^G/G^/J20 mice was among the reasons for the recovery of spatial memory behaviours in J20 mice. We acknowledge, however, changes in other brain areas involved in these behaviours may also be contributing to the phenotype rescue.

### Abolishing unedited GluA2(Q) expression does not affect the seizure phenotype of J20 mice

AD patients have a higher incidence of seizures than the general population [79, 80], but the mechanisms causing this are not yet clear. Spontaneous seizures and susceptibility to seizure-inducing drugs are observed in J20 mice [80, 81, 118], making this line a useful model to investigate the causes of this phenotype [81, 119]. Recently, it was suggested that severe developmental and epileptic encephalopathy (DEE) may be caused by a mutation in ADAR2 that leads to hypoediting of GluA2 at the Q/R site [120]. In support of this, a recent study has identified several patients with neurodevelopmental disorders including DEE all of which displayed de novo mutations in *GRIA2* including one patient with a mutation in the Q/R editing site who displayed a seizure phenotype [121]. Furthermore, we [58] and others [56, 57, 99] have found that mice genetically engineered to express higher levels of unedited GluA2(Q) experience spontaneous seizures and are susceptible to kainic acid-induced seizures. Given this evidence, we therefore hypothesised that GluA2(Q) may be mechanistically contributing to seizure phenotypes in J20 mice.

Surprisingly, however, we did not observe any difference in spontaneous seizures at 40+ weeks of age, or in susceptibility to kainic acid-induced seizures in Gria2^G/G^/J20 compared to J20 mice at 22 weeks of age, suggesting the mechanistic drivers of seizure and seizure-susceptibility are independent of unedited GluA2(Q) in J20 mice. Although the mechanism of seizures in J20 mice and in humans with AD is unknown, some prior studies have reported reductions in EEG spikes in J20 mice using different approaches. Sanchez et al., reported that the drug Levetiracetam reduced EEG spikes in J20 mice [104]. The mechanisms of action for Levetiracetam are not yet clear but may be related to binding the synaptic vesicle protein 2A (SV2A). Another study reported that enhancing the levels of Na_v_1.1 (a voltage-gated sodium channel subunit expressed by inhibitory parvalbumin cells, but with decreased expression in J20 mice), reduces epileptiform activity in J20 mice [80]. This suggests a loss of Na_v_1.1 expression in parvalbumin cells may be contributing to seizure phenotypes in J20 mice. Although these prior studies highlight several mechanisms that may account for the seizure phenotype of J20 mice independently of GluA2(Q), it is possible that our results may also be explained by the lack of reduction in Aβ-pathology in our study. Others have suggested Aβ-pathology is a direct cause of seizure-activity in J20 mice [81, 106, 118], possibly via a coordinated Aβ-tau-Fyn effect on network function. This was supported by the finding that reducing endogenous tau expression reduces EEG spikes and spontaneous seizures [118].

Although unedited GluA2(Q) does not appear to be contributing to the seizure phenotype of J20 mice in the ages we examined, we cannot conclusively rule out that it is contributing to seizure phenotypes in human AD, or other neurological conditions. Use of a Ca^2+^-permeable AMPAR antagonist in experiments investigating seizure vulnerability would be useful to exclude these receptors (be it GluA2(Q) or GluA2-lacking) in this phenotype. Importantly, although we did not observe a decline in seizure activity in Gria2^G/G^/J20 mice, this did not preclude improvements in spatial reference and working memory, suggesting these cognitive impairments may be independent of seizure activity.

### ADAR2 downregulation as a potential cause of RNA editing deficiencies in AD

ADAR2 has numerous editing targets and non-editing functions [122]. Importantly, KO of ADAR2 is lethal but can be rescued by exonically encoding the edited CGG codon in *Gria2* [35, 123], suggesting that GluA2(Q) expression is the most devastating consequence of reduced ADAR2 activity. Changes in the efficiency of editing at the Q/R site of GluA2 have been reported in several human AD brain regions including the prefrontal cortex [51], temporal lobe [21] and hippocampus [50, 52], strongly suggesting the proportion of AMPARs containing unedited GluA2(Q) are increased in AD. Our study has identified a putative role for unedited GluA2(Q) in the development of AD-related pathologies, but the mechanisms driving this are not yet clear. The most logical cause of GluA2 Q/R site hypoediting in AD would be a change in the expression, activity, or cellular localisation of ADAR2 [124–126]. In line with this hypothesis, we report a significant downregulation of ADAR2 protein expression in the hippocampus of both J20 mice and another AD mouse model, 5XFAD, which we propose may be driving hypoediting of the Q/R site of GluA2.

While GluA2 Q/R site editing has been shown to be reduced in human AD brains, studies of ADAR2 mRNA expression have shown mixed results. In one study, a reduction of ADAR2 mRNA expression was observed in the caudate nucleus of AD patients, but not the hippocampus [50]. Another study found a slight, but non-significant decline in ADAR2 mRNA levels within the hippocampus of patients with late-onset AD [21]; however, a significant increase in the expression of ADAR3 was also reported, which the authors speculate may be competitively inhibiting ADAR1 and ADAR2 binding to their target transcripts, thereby driving widespread hypoediting. Contradictory to these findings, one study found an increase in the expression of ADAR2 mRNA in the temporal lobe of AD patients, however there was significant hypoediting of the GluA2 Q/R site in the same region [52]. Another study found no differences in ADAR2 mRNA expression in blood derived from human AD patients, although the relationship between brain-derived ADAR2 and ADAR2 measured in blood is unclear [53]. More recently, however, mRNA expression of ADAR2 was found to be significantly reduced in the prefrontal cortex of AD patients [49]. Thus, while studies assessing ADAR2 mRNA expression are inconsistent, evidence of ADAR2 regulation at the protein level is beginning to emerge. In this context, it is notable that a recent study observed mislocalisation of ADAR2, and widespread alterations to ADAR2 editing substrates in human brain tissue from ALS, FTD and severe AD patients [42], suggesting it may not simply be a matter of changes in ADAR2 gene expression that could cause editing deficiencies in AD, but also ADAR2 protein mislocalisation. Indeed, other factors regulating ADAR2 activity, but not its gene expression, include binding to peptidyl-prolyl cis-trans isomerase NIMA-interacting 1 (PIN1) [127], which is required for ADAR2 nuclear localisation, and the ability of ADAR2 to self-edit [128]. Both of these processes have been implicated in neurological disorders [41, 129] and, moreover, two PIN1 single nucleotide polymorphisms (SNPs) are considered risk factors for sporadic AD [130].

Collectively, studies strongly suggest ADAR2 may be dysregulated in human AD, but more work clearly needs to be done to establish how ADAR2 expression, activity and cellular localisation change in different brain regions, cell types, and at different stages of AD. It is also unclear what upstream mechanisms may be causing ADAR2 dysregulation in AD, and what the implications of ADAR2 dysregulation may be for ADAR2-targeted editing sites other than the Q/R site of GluA2. It is worth noting that decreased expression of ADAR2 has been observed in other neurodegenerative diseases, including ALS [131] and forebrain ischemia [44] and the resulting impairment of GluA2 Q/R site editing is thought to be linked to neuronal death in these conditions. Thus, downregulation of ADAR2 activity may be upstream of neuronal degeneration in other neurological conditions.

### The role of GluA2(Q) in regulating dendritic spines

Regulation of dendritic spines is known to be a physiologically dynamic process ranging from spinogenesis and spine elimination to spine remodelling and is critical to both normal development and cognitive function [76, 132]. An unexpected and important observation of our study is that spine regulation may involve GluA2 Q/R site editing.

Previous studies have indicated that downregulation of GluA2, and thus elevated Ca^2+^-permeable AMPARs, can alter maturation of dendritic arborisation during critical development periods, and that GluA2 levels within spines can be predictive of spine behaviour in vivo [133–135]. In AD models, Ca^2+^-permeable AMPARs are known to lead to synaptic weakening, and it has been postulated this can lead to increased tau-hyperphosphorylation, escalating the disease cycle. Conversely, in vitro evidence suggests that the overexpression of GluA2 induces a higher density of longer and wider spines in neurons [136] However, although it has long been known that RNA editing at the GluA2 Q/R site is evolutionarily conserved, it is unclear why GluA2(R) is not simply encoded in the genome and for what purpose a complex process of editing is required instead [137].

We unexpectedly observed that Gria2^G/G^ and Gria2^G/G^/J20 mice have increased dendritic spine density compared to WT mice, suggesting GluA2 Q/R site editing may regulate dendritic spine growth. Previous studies have suggested that GluA2(Q) may be important for learning and memory [138], an idea that would fit neatly with our results suggesting GluA2(Q) as a regulator of spine growth. Our finding is the first demonstration of any difference in mice with genetically-encoded edited GluA2(R), with prior work failing to find any differences compared to WT animals [35, 123]. Additional studies in our mice, such as tests of sociability, sensory and cognitive ability, as well as investigations into spine types and signalling, might reveal alterations indicating a functional role for RNA editing at the GluA2 Q/R site in regulating dendritic spine dynamics in the normal adult mammalian brain.

## Conclusions

Prior research indicates that unedited GluA2(Q) can be incorporated into mature AMPARs and cause synapse and neuronal pathology through excitotoxicity [58, 61]. Therefore, alterations in unedited GluA2(Q) expression are likely to cause a pathological overactivation of AMPARs, leading to excitotoxic cell death [61], an idea that fits with the Ca^2+^ hypothesis of AD [139]. Taken together with the data presented here, we propose that expression of unedited GluA2(Q) is aetiologically linked to synaptic signalling deficits, dendritic and neuronal pathology, and memory impairments in J20 mice.

Our findings have three clear implications. First, they suggest aberrant RNA editing at the Q/R site of GluA2 may be a novel mechanism of synapse loss and neurodegeneration in AD. Second, they raise the prospect that therapeutically targeting GluA2 Q/R site RNA editing may improve neuronal survival and memory in AD. Finally, they suggest that RNA editing may regulate dendritic spines in healthy brains. Thus, we propose that RNA editing at the Q/R site of GluA2 acts as an epigenetic switch regulating dendritic spines in health and disease.

### Supplementary Information

**Additional file 1: Sup Figure 1.** Generation of *Gria2*^tm1BViss^ mice and GluA2 Q/R site editing efficiency analysis. (a) Schematic representation of the GluA2 WT allele, the targeted GluA2^G/G/neo^ allele and the targeted GluA2^G/G^ allele, after the removal of the floxed neo cassette by Cre-mediated recombination in ES cells. Exons 10, 11 and 12 are shown (black boxes). Black arrows indicate loxP sites. The position of the adenosine to guanine mutation is indicated in red. (b) DNA sequencing of WT and *Gria2*^tm1BViss^ mice confirmed the single adenosine to guanine mutation in homozygous mice. (c) Genotype analysis of WT, GluA2^G/-^ and GluA2^G/G^ mice by PCR shows a band at 200 bp in WT, two bands at 200 bp and 250 bp in heterozygous mice and a single band at 250bp in homozygous mice. (d) *BbvI* digestion assay. Schematic representation of the GluA2 mRNA *Bbv1* digestion assay shows 2 bands produced for edited GluA2 templates (225bp and 68bp) and 3 bands for unedited GluA2 template (144 bp, 81bp and 68 bp). Representative image and quantification of Bbv1 digestions revealed GluA2^G/G^ mice exhibit 0% unedited GluA2, whereas WT animals exhibit 0.44% unedited GluA2 in the hippocampus (*n* = 3/genotype). Each value represents the mean ± the SD.

**Additional file 2: Sup Figure 2.** Body weight measurement. J20 and GluA2^G/G^/J20 mice display reduced body weight when compared to WT and GluA2^G/G^ (Kruskal-Wallis = 19.40, *p* = 0.0002; *n’s*: WT = 34, GluA2^G/G ^= 26, J20 = 30, GluA2^G/G^/J20 = 31). Each value represents the mean ± the SD. *p < 0.05, **p < 0.01.

**Additional file 3: Sup Figure 3.** Upon AMPA stimulation, no cobalt uptake occurred in the presence of Ca^2+^-permeable antagonists, GYKI or NBQX, in acute slices taken from mice of all genotypes demonstrating cobalt uptake can only occur through Ca^2+^-permeable AMPARs.

**Additional file 4: Sup Figure 4.** AMPAR surface expression and complex formulation. (a-b) BS^3^ crosslinking of surface AMPARs alters their molecular weight enabling the discrimination of surface-crosslinked versus intracellular-non crosslinked AMPARs. No changes were found to the ratio of surface to intracellular GluA2 in the (a) whole hippocampus or in (b) hippocampal subregions: CA1, CA3 or DG (*n’s* for a: WT = 7, GluA2^G/G^= 8, J20 = 8, GluA2^G/G^/J20 = 8; *n’s* for b: WT = 4 for CA1 and DG and 5 for CA3, GluA2^G/G^= 4 for CA1 and DG and 5 for CA3, J20 = 5 for CA1 and CA3 and 4 for DG, GluA2^G/G^/J20 = 5 for CA1 and CA3 and 4 for DG; CA1 ANOVA: *F*_(3,14)_  =  1.607 *p* = 0.232; CA3 ANOVA: *F*_(3,16)_  =  1.403 *p* = 0.278; DG ANOVA: *F*_(3,12)_  =  1.262 *p* = 0.24). *N’s* represent averaged normalised values per immunoblot. Total protein expression of (c) GluA2 and (d) GluA2/3 in the hippocampus showed no differences between any of the genotypes (*n* = 6/genotype except for (c) where GluA2^G/G^= 5 and GluA2^G/G^/J20 = 5; GluA2 ANOVA: *F*_(3,18)_  =  1.068 *p* = 0.39; GluA2/3 ANOVA: *F*_(3,20)_  =  0.51 *p* = 0.68). (e) Co-immunoprecipitation of AMPAR subunits demonstrated none of the genotypes showed any alterations to their AMPA receptor composition within the hippocampus (unbound fraction shown; *n’s*: WT = 4, GluA2^G/G^= 3, J20 = 4, GluA2^G/G^/J20 = 4). For example, the first column of image in top left shows 100% of GluA1 remained in the unbound fraction when IP’ed against the IgG control, followed by <5% of GluA1 remaining in the unbound fraction when IP’ed against GluA1, followed by 12% of GluA1 remaining in the unbound fraction when IP’ed against GluA2, etc. Each value represents the mean ± the SD.

**Additional file 5: Sup Figure 5.** ADAR2 downregulation. ADAR2 expression is downregulated as shown in immunoassays from (a) J20 mice and (b) 5xFAD mice as compared to WT littermates (t-test = 2.68, *p* < 0.028; *n* = 5/genotype; t-test = 2.95, *p* = 0.021; *n’s*: WT = 5 and 5xFAD = 4). B shows interpolated blots as well as example electropherograms (EPG) used for analysis. Each value represents the mean ± the SD. *p<0.05.

**Additional file 6: Sup Figure 6.** Genetically encoding GluA2(R) in J20 mice does not prevent neuroinflammation. (a) Representative images and stereological quantification revealed no changes were observed in the population of GFAP^+^ astrocytes within the CA1 and CA3 hippocampal regions (CA1 ANOVA *F*_(3,16)_  =  0.39 *p* = 0.76 and CA3 ANOVA *F*_(3,16)_  =  2.27 *p* = 0.12; *n* = 5/genotype). (b) Representative images and stereological quantification of CD68^+^ microglia in the hippocampus demonstrated a significant increase in both J20 and GluA2^G/G^/J20 animals when compared to WT and GluA2^G/G^ mice (Kruskal-Wallis = 21.52 *p* <0.0001; *n* = 8 for WT and *n* = 7 for all other genotypes). (c) An ELISA revealed TNF protein expression in the hippocampus is significantly upregulated in J20 mice compared to WTs and this was not prevented in the GluA2^G/G^/J20 mice (ANOVA *F*_(3,28)_  =  8.65 *p* <0.001; *n* = 8/genotype). Each value represents the mean ± the SD. *p<0.05, **p<0.01, ***p<0.001.

**Additional file 7: Sup. Figure 7.** Synaptic transmission and short-term plasticity. (a) Input/output ratios and (b) paired pulse facilitation remain unchanged in all genotypes (*n’s*: WT = 24 slices (a) and 26 slices (b) from 9 mice, GluA2^G/G^ = 21 slices from 7 mice, J20 = 24 slices from 7 mice, GluA2^G/G^/J20 = 13 slices from 7 mice; input/output ratio two-way RM ANOVA: genotype effect *F*_(3,81)_  =  0.386, *p* = 0.76; PPF two-way RM ANOVA: genotype effect *F*_(3,81)_  =  0.386, *p* = 0.76). Each value represents the mean ± the SEM.

**Additional file 8: Sup Figure 8.** Behavioural assessment. (a-c) The total number of arm entries made in the Y-maze (a) as well as the time spent exploring the novel object in the recognition task (b) and the total time spent in the working memory version of the RAM (c) was not different between any of the genotypes indicating that hyperactivity did not affect animals’ ability to perform in these tests (*n’s* for a: WT = 24, GluA2^G/G^ = 20, J20 = 24, GluA2^G/G^/J20 = 22; *n’s* for b: WT = 25, GluA2^G/G^ = 21, J20 = 28, GluA2^G/G^/J20 = 22; *n’s* for c: WT = 14, GluA2^G/G^ = 10, J20 = 10, GluA2^G/G^/J20 = 11; Y-maze entries ANOVA: *F*_(3,8)_  =  2.271, *p* = 0.09; Object Recognition ANOVA: *F*_(3,92)_  =  0.754, *p* = 0.52; RAM total time two-way RM ANOVA: genotype effect *F*_(3,41)_  =  1.907, *p* = 0.14). (d) Total distance travelled in the open field test revealed both J20 and GluA2^G/G^/J20 mice displayed significantly more hyperactivity than WT and GluA2^G/G^ mice (ANOVA *F*_(3,88)_  =  18.90, *p* <0.0001; *n’s*: WT = 26, GluA2^G/G^ = 20, J20 = 27, GluA2^G/G^/J20 = 19). (e) The ratio of open arm to total arm entries in the elevated plus maze showed a trend towards more open arm entries in J20 animals and a recovery of this in GluA2^G/G^/J20 mice (Welch’s ANOVA *W*_(3,29.15)_ = 2.12, *p* = 0.12; *n’s*: WT = 15, GluA2^G/G^ = 13, J20 = 16, GluA2^G/G^/J20 = 14). (f) Both J20 and GluA2^G/G^/J20 mice displayed an increased latency to fall from the rotarod on Day 1 of testing compared to WT and GluA2^G/G^ mice, however, no differences were observed between any of the genotypes on testing days 2 and 3 indicating a normal motor learning ability for all groups across the testing period (RM ANOVA for time: *F*_(2,232)_  =  15.16, *p* < 0.0001; for genotype: *F*_(3,116)_  =  4.96, *p* < 0.01; *n’s*: WT = 33, GluA2^G/G^ = 24, J20 = 36, GluA2^G/G^/J20 = 27). Each value represents the mean ± the SD for bar graphs and SEM for line graphs. *p < 0.05, **p < 0.01 ***p < 0.001, ****p < 0.0001.

## Data Availability

The datasets used and/or analysed during the current study as well as the *Gria2*^tm1BViss^ mice are available from the corresponding author on reasonable request.

## Abbreviations

6E10: Anti-β-Amyloid clone number 6E10
A: Adenosine
Aβ: Amyloid-β
aCSF: Artificial cerebrospinal fluid
AD: Alzheimer’s disease
ADAR1: Adenosine deaminases acting on RNA 1
ADAR2: Adenosine deaminases acting on RNA 2
ADAR3: Adenosine deaminases acting on RNA 3
AEC: Animal ethics committee
AGS: Aicardi-Goutieres syndrome
AMPAR: α-Amino-3-hydroxy-5-methyl-4-Isoxazolepropionic acid receptor
ANOVA: Analysis of variance
APP: Amyloid precursor protein
C9orf72: Chromosome 9 open reading frame 72
Ca^2+^: Calcium ions
CD-68: Cluster of differentiation 68
Co^2+^: Cobalt ions
CoCl2: Cobalt chloride
DEE: Developmental and epileptic encephalopathy
DNA: Deoxyribonucleic acid
ECoG: Electrocorticography
EEG: Electroencephalogram
EPG: Electropherogram
EPM: Elevated plus maze
EPSC: Excitatory postsynaptic current
ES: Embryonic stem
fEPSP: Field excitatory postsynaptic potential
FTD: Frontotemporal dementia
G: Guanine
GFAP: Glial fibrillary acidic protein
GluA2: Glutamate receptor subunit 2
GluA3: Glutamate receptor subunit 3
GRIA2: Glutamate ionotropic receptor AMPA type subunit 2
GYKI: 1-(4-Aminophenyl)-4-methyl-7,8-methylenedioxy-5H-2,3-benzodiazepine hydrochloride
I/V: Current/voltage
IVC: Individually ventilated cage
KA: Kainic acid
KO: Knock-out
LTP: Long-term potentiation
MND: Motor neuron disease
MRI: Magnetic resonance imaging
mV: Millivolt
NASPM: 1-Naphthylacetyl spermine
Nav1.1: Voltage-gated sodium channel type I alpha subunit
NBQX: 2,3-Dioxo-6-nitro-1,2,3,4-tetrahydrobenzo[f]quinoxaline-7-sulfonamide disodium salt
NeuN: Neuronal nuclei
OFT: Open field test
pA: Picoampere
PCR: Polymerase chain reaction
PSEN1: Presenilin 1
PSEN2: Presenilin 2
Q: Glutamine
RAM: Radial arm maze
RNA: Ribonucleic acid
R: Arginine
SD: Standard deviation
SEM: Standard error of the mean
SV2A: Synaptic vesicle protein 2A
TBS: Theta burst stimulation
TNF-α: Tumor necrosis factor α
WT: Wildtype
