# Supplementary figures and images for: The Q/R editing site of AMPA receptor GluA2 subunit acts as an epigenetic switch regulating dendritic spines, neurodegeneration and cognitive deficits in Alzheimer’s disease
Source: Mol Neurodegener. 2023 Sep 28;18:65. doi: 10.1186/s13024-023-00632-5 (PMC10537207; doi:10.1186/s13024-023-00632-5)

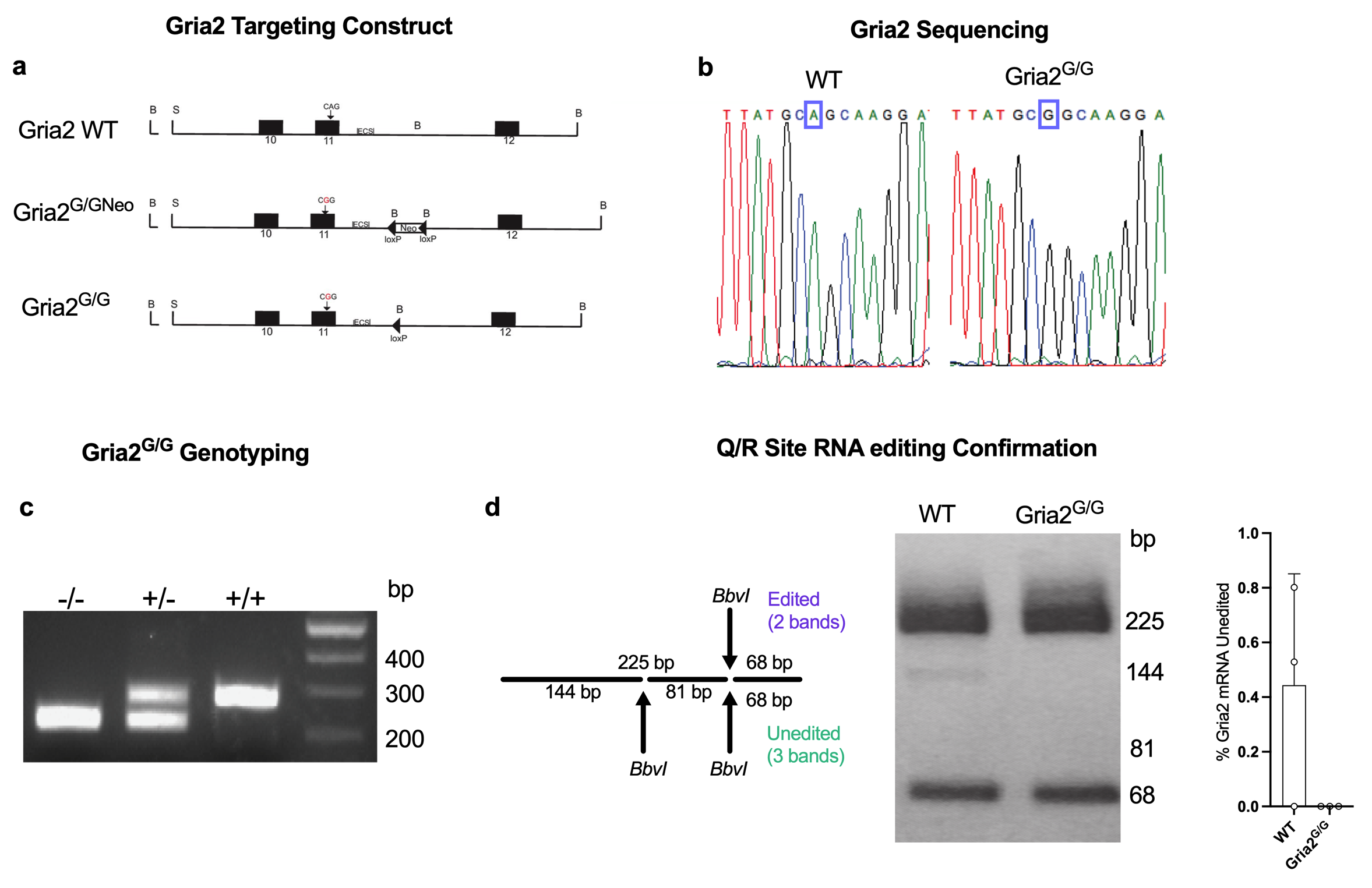

Supplement: Supplementary file 1 — Additional file 1: Sup Figure 1. Generation of Gria2tm1BViss mice and GluA2 Q/R site editing efficiency analysis. (a) Schematic representation of the GluA2 WT allele, the targeted GluA2G/G/neo allele and the targeted GluA2G/G allele, after the removal of the floxed neo cassette by Cre-mediated recombination in ES cells. Exons 10, 11 and 12 are shown (black boxes). Black arrows indicate loxP sites. The position of the adenosine to guanine mutation is indicated in red. (b) DNA sequencing of WT and Gria2tm1BViss mice confirmed the single adenosine to guanine mutation in homozygous mice. (c) Genotype analysis of WT, GluA2G/- and GluA2G/G mice by PCR shows a band at 200 bp in WT, two bands at 200 bp and 250 bp in heterozygous mice and a single band at 250bp in homozygous mice. (d) BbvI digestion assay. Schematic representation of the GluA2 mRNA Bbv1 digestion assay shows 2 bands produced for edited GluA2 templates (225bp and 68bp) and 3 bands for unedited GluA2 template (144 bp, 81bp and 68 bp). Representative image and quantification of Bbv1 digestions revealed GluA2G/G mice exhibit 0% unedited GluA2, whereas WT animals exhibit 0.44% unedited GluA2 in the hippocampus (n = 3/genotype). Each value represents the mean ± the SD. [file 13024_2023_632_MOESM1_ESM.tiff]

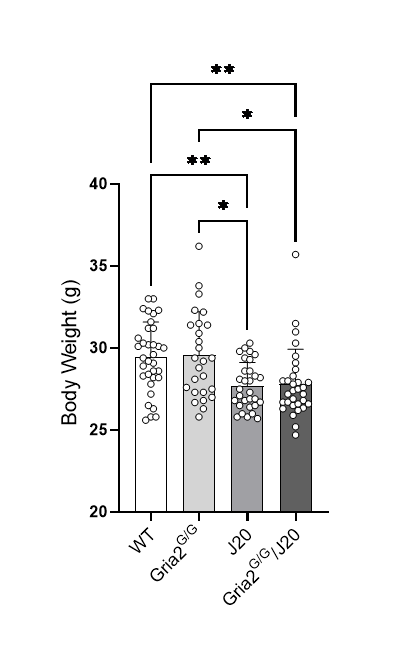

Supplement: Supplementary file 2 — Additional file 2: Sup Figure 2. Body weight measurement. J20 and GluA2G/G/J20 mice display reduced body weight when compared to WT and GluA2G/G (Kruskal-Wallis = 19.40, p = 0.0002; n’s: WT = 34, GluA2G/G = 26, J20 = 30, GluA2G/G/J20 = 31). Each value represents the mean ± the SD. *p < 0.05, **p < 0.01. [file 13024_2023_632_MOESM2_ESM.tif]

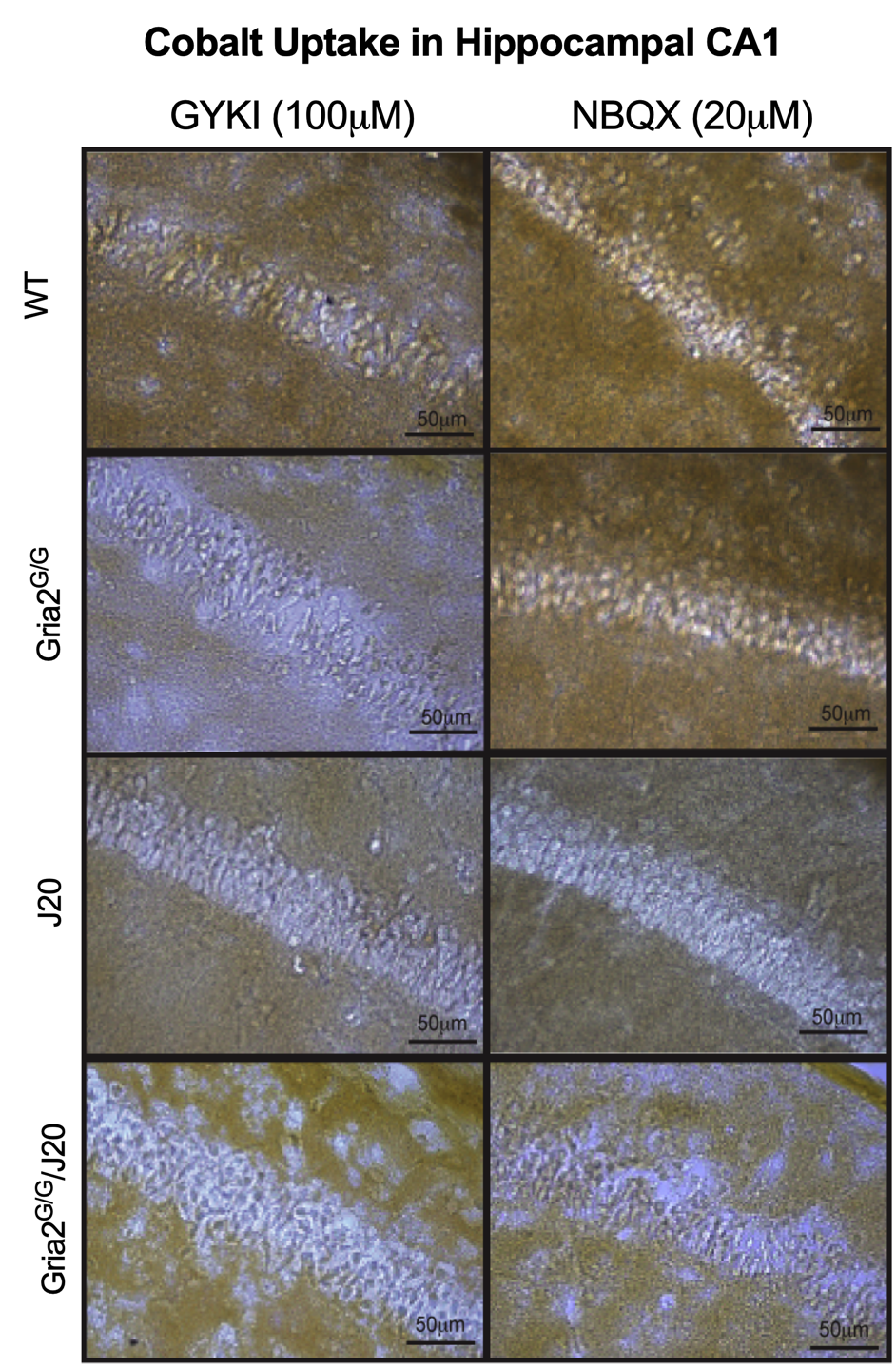

Supplement: Supplementary file 3 — Additional file 3: Sup Figure 3. Upon AMPA stimulation, no cobalt uptake occurred in the presence of Ca2+-permeable antagonists, GYKI or NBQX, in acute slices taken from mice of all genotypes demonstrating cobalt uptake can only occur through Ca2+-permeable AMPARs. [file 13024_2023_632_MOESM3_ESM.tiff]

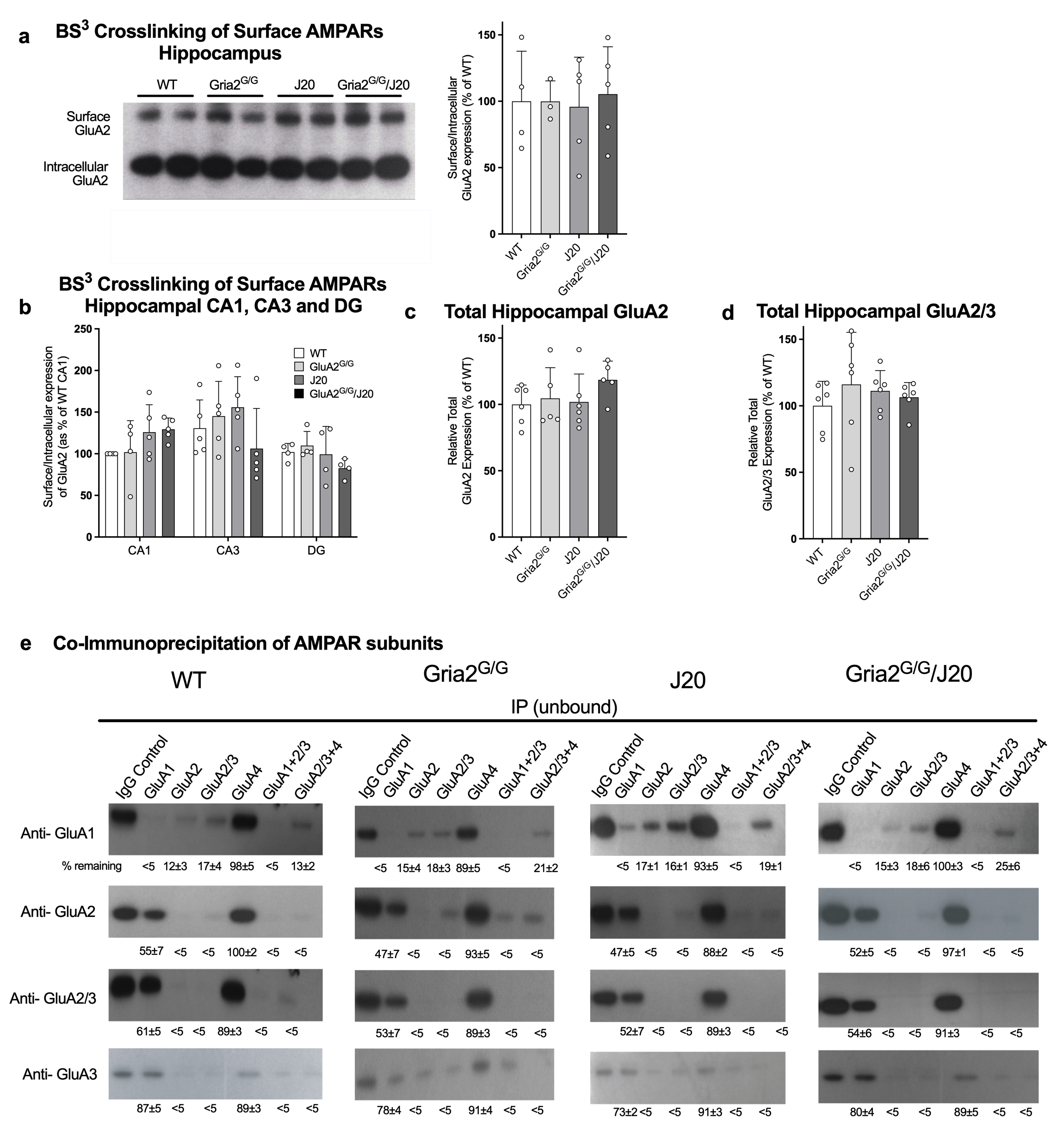

Supplement: Supplementary file 4 — Additional file 4: Sup Figure 4. AMPAR surface expression and complex formulation. (a-b) BS3 crosslinking of surface AMPARs alters their molecular weight enabling the discrimination of surface-crosslinked versus intracellular-non crosslinked AMPARs. No changes were found to the ratio of surface to intracellular GluA2 in the (a) whole hippocampus or in (b) hippocampal subregions: CA1, CA3 or DG (n’s for a: WT = 7, GluA2G/G= 8, J20 = 8, GluA2G/G/J20 = 8; n’s for b: WT = 4 for CA1 and DG and 5 for CA3, GluA2G/G= 4 for CA1 and DG and 5 for CA3, J20 = 5 for CA1 and CA3 and 4 for DG, GluA2G/G/J20 = 5 for CA1 and CA3 and 4 for DG; CA1 ANOVA: F(3,14) = 1.607 p = 0.232; CA3 ANOVA: F(3,16) = 1.403 p = 0.278; DG ANOVA: F(3,12) = 1.262 p = 0.24). N’s represent averaged normalised values per immunoblot. Total protein expression of (c) GluA2 and (d) GluA2/3 in the hippocampus showed no differences between any of the genotypes (n = 6/genotype except for (c) where GluA2G/G= 5 and GluA2G/G/J20 = 5; GluA2 ANOVA: F(3,18) = 1.068 p = 0.39; GluA2/3 ANOVA: F(3,20) = 0.51 p = 0.68). (e) Co-immunoprecipitation of AMPAR subunits demonstrated none of the genotypes showed any alterations to their AMPA receptor composition within the hippocampus (unbound fraction shown; n’s: WT = 4, GluA2G/G= 3, J20 = 4, GluA2G/G/J20 = 4). For example, the first column of image in top left shows 100% of GluA1 remained in the unbound fraction when IP’ed against the IgG control, followed by <5% of GluA1 remaining in the unbound fraction when IP’ed against GluA1, followed by 12% of GluA1 remaining in the unbound fraction when IP’ed against GluA2, etc. Each value represents the mean ± the SD. [file 13024_2023_632_MOESM4_ESM.tiff]

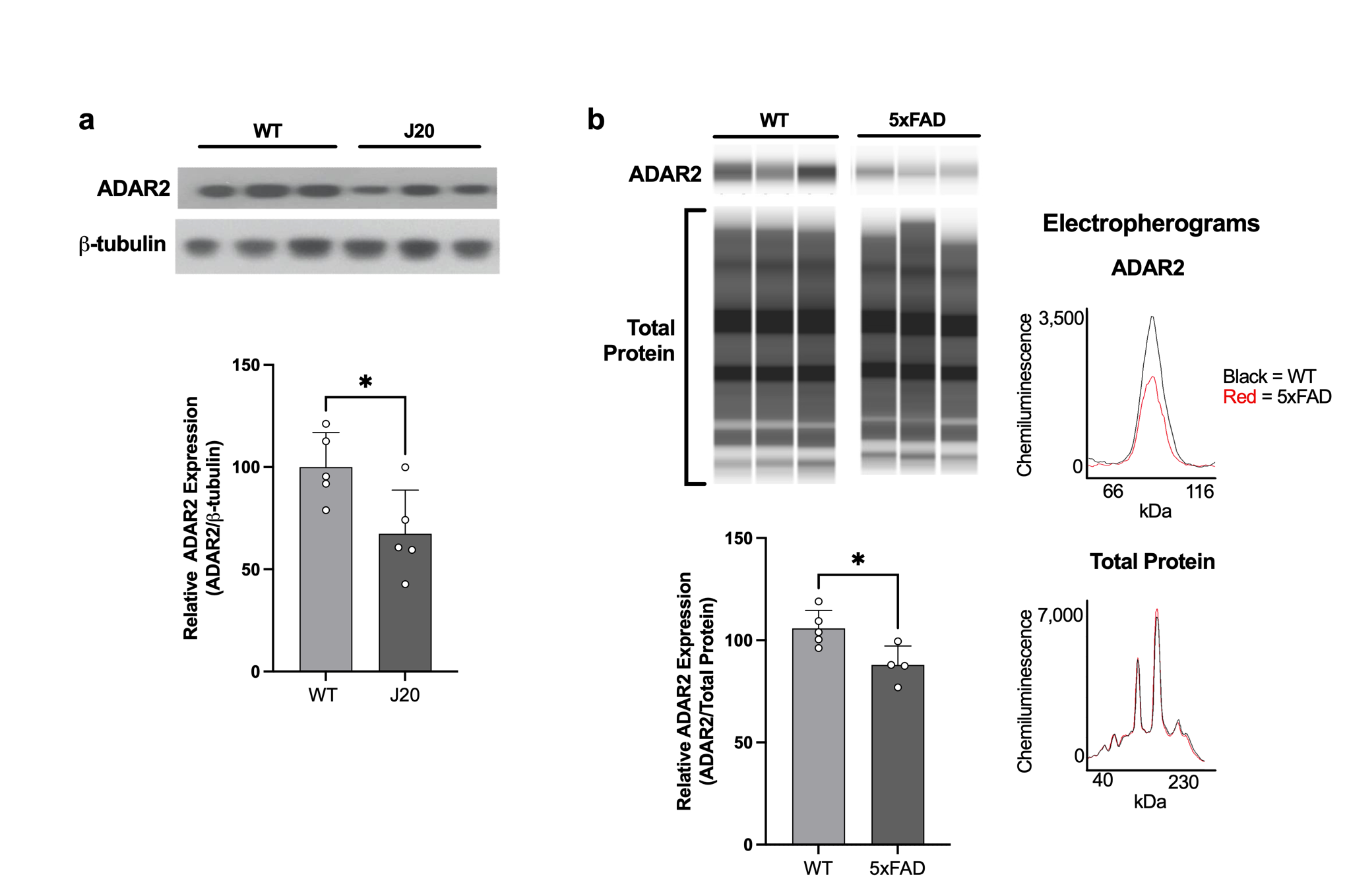

Supplement: Supplementary file 5 — Additional file 5: Sup Figure 5. ADAR2 downregulation. ADAR2 expression is downregulated as shown in immunoassays from (a) J20 mice and (b) 5xFAD mice as compared to WT littermates (t-test = 2.68, p < 0.028; n = 5/genotype; t-test = 2.95, p = 0.021; n’s: WT = 5 and 5xFAD = 4). B shows interpolated blots as well as example electropherograms (EPG) used for analysis. Each value represents the mean ± the SD. *p<0.05. [file 13024_2023_632_MOESM5_ESM.tiff]

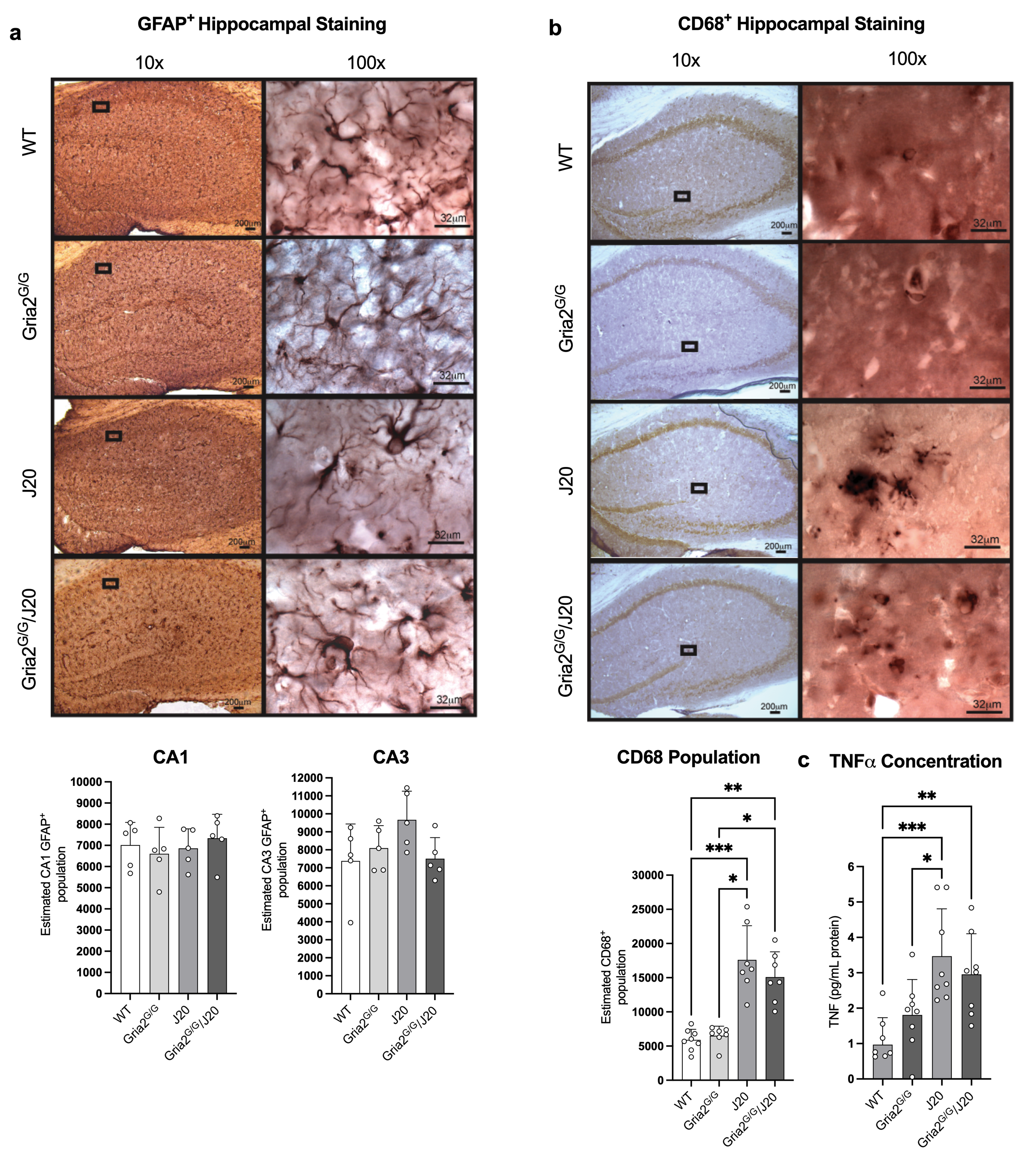

Supplement: Supplementary file 6 — Additional file 6: Sup Figure 6. Genetically encoding GluA2(R) in J20 mice does not prevent neuroinflammation. (a) Representative images and stereological quantification revealed no changes were observed in the population of GFAP+ astrocytes within the CA1 and CA3 hippocampal regions (CA1 ANOVA F(3,16) = 0.39 p = 0.76 and CA3 ANOVA F(3,16) = 2.27 p = 0.12; n = 5/genotype). (b) Representative images and stereological quantification of CD68+ microglia in the hippocampus demonstrated a significant increase in both J20 and GluA2G/G/J20 animals when compared to WT and GluA2G/G mice (Kruskal-Wallis = 21.52 p <0.0001; n = 8 for WT and n = 7 for all other genotypes). (c) An ELISA revealed TNF protein expression in the hippocampus is significantly upregulated in J20 mice compared to WTs and this was not prevented in the GluA2G/G/J20 mice (ANOVA F(3,28) = 8.65 p <0.001; n = 8/genotype). Each value represents the mean ± the SD. *p<0.05, **p<0.01, ***p<0.001. [file 13024_2023_632_MOESM6_ESM.tiff]

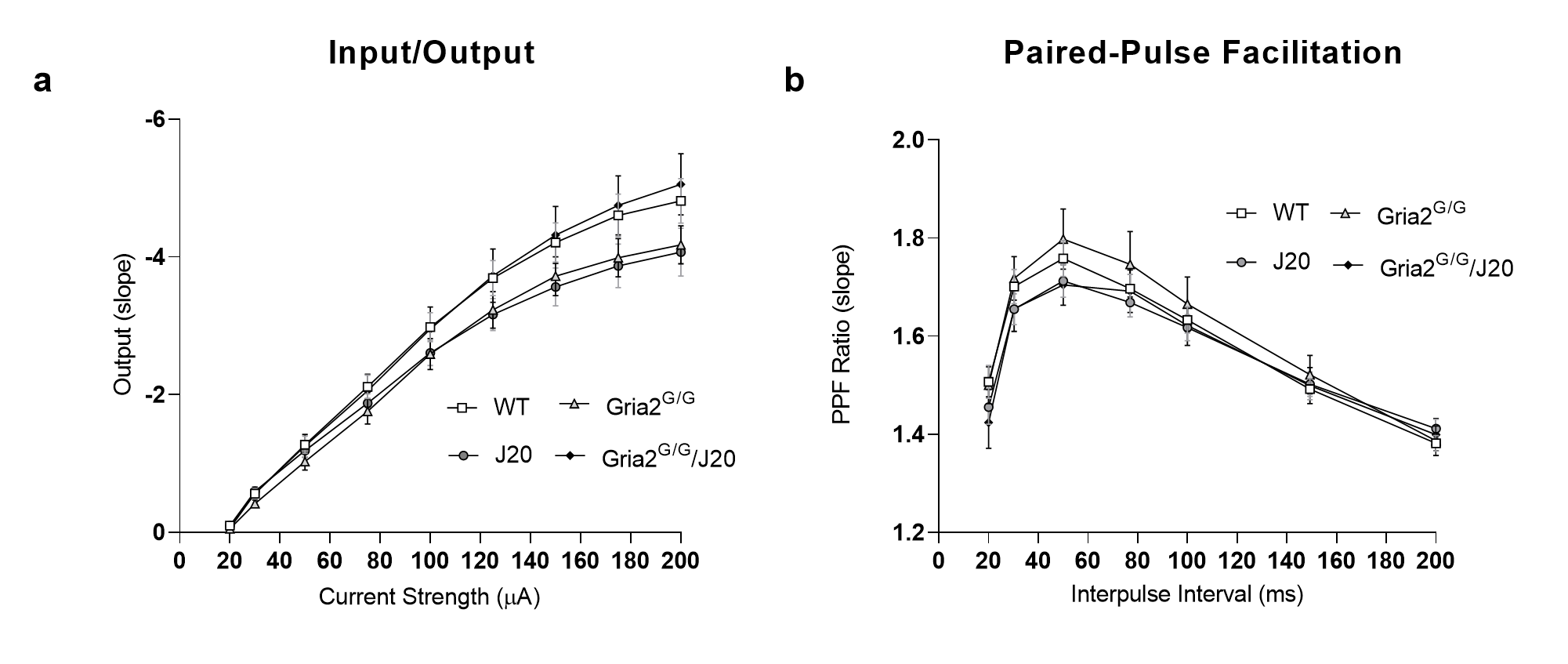

Supplement: Supplementary file 7 — Additional file 7: Sup. Figure 7. Synaptic transmission and short-term plasticity. (a) Input/output ratios and (b) paired pulse facilitation remain unchanged in all genotypes (n’s: WT = 24 slices (a) and 26 slices (b) from 9 mice, GluA2G/G = 21 slices from 7 mice, J20 = 24 slices from 7 mice, GluA2G/G/J20 = 13 slices from 7 mice; input/output ratio two-way RM ANOVA: genotype effect F(3,81) = 0.386, p = 0.76; PPF two-way RM ANOVA: genotype effect F(3,81) = 0.386, p = 0.76). Each value represents the mean ± the SEM. [file 13024_2023_632_MOESM7_ESM.tif]

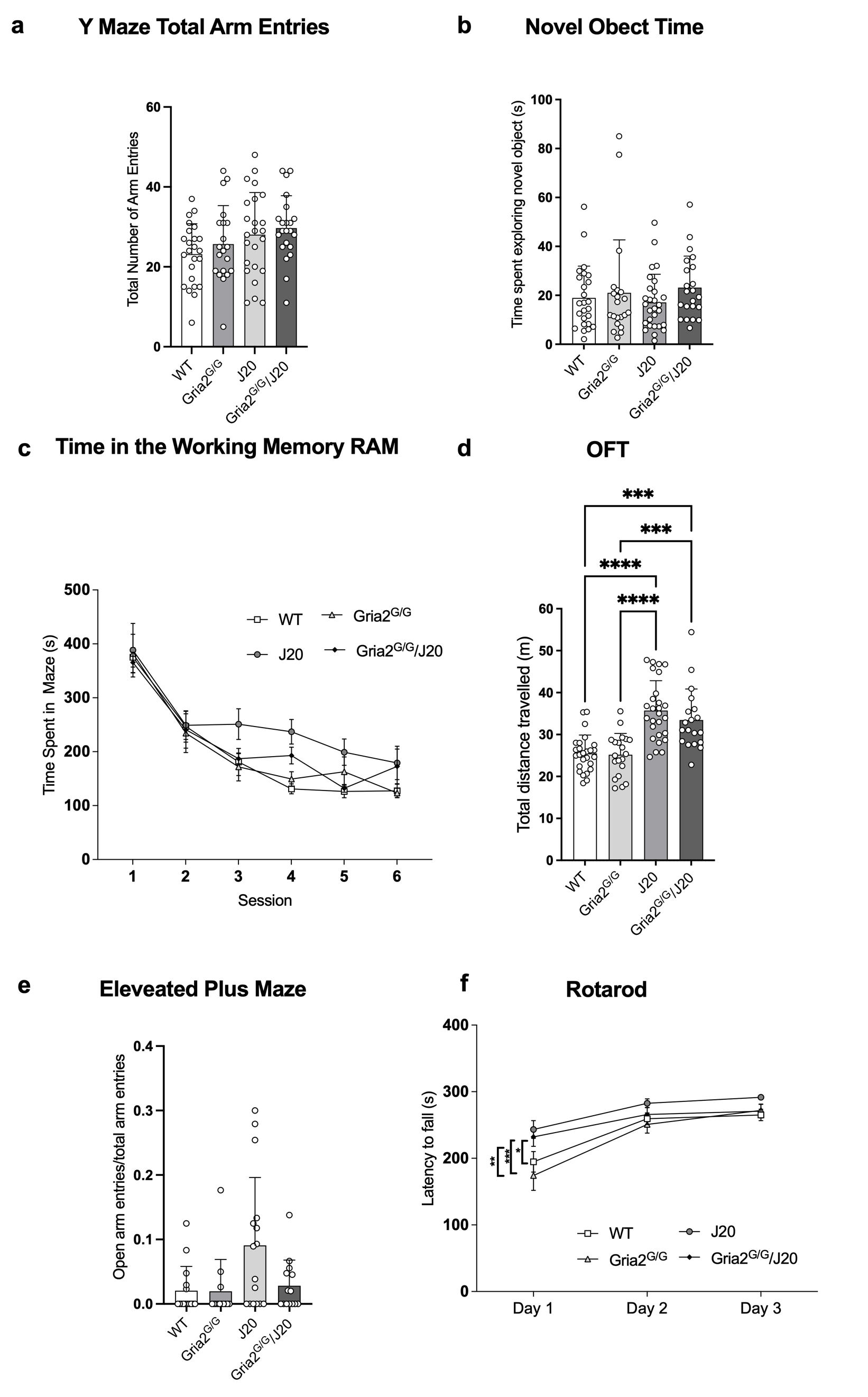

Supplement: Supplementary file 8 — Additional file 8: Sup Figure 8. Behavioural assessment. (a-c) The total number of arm entries made in the Y-maze (a) as well as the time spent exploring the novel object in the recognition task (b) and the total time spent in the working memory version of the RAM (c) was not different between any of the genotypes indicating that hyperactivity did not affect animals’ ability to perform in these tests (n’s for a: WT = 24, GluA2G/G = 20, J20 = 24, GluA2G/G/J20 = 22; n’s for b: WT = 25, GluA2G/G = 21, J20 = 28, GluA2G/G/J20 = 22; n’s for c: WT = 14, GluA2G/G = 10, J20 = 10, GluA2G/G/J20 = 11; Y-maze entries ANOVA: F(3,8) = 2.271, p = 0.09; Object Recognition ANOVA: F(3,92) = 0.754, p = 0.52; RAM total time two-way RM ANOVA: genotype effect F(3,41) = 1.907, p = 0.14). (d) Total distance travelled in the open field test revealed both J20 and GluA2G/G/J20 mice displayed significantly more hyperactivity than WT and GluA2G/G mice (ANOVA F(3,88) = 18.90, p <0.0001; n’s: WT = 26, GluA2G/G = 20, J20 = 27, GluA2G/G/J20 = 19). (e) The ratio of open arm to total arm entries in the elevated plus maze showed a trend towards more open arm entries in J20 animals and a recovery of this in GluA2G/G/J20 mice (Welch’s ANOVA W(3,29.15) = 2.12, p = 0.12; n’s: WT = 15, GluA2G/G = 13, J20 = 16, GluA2G/G/J20 = 14). (f) Both J20 and GluA2G/G/J20 mice displayed an increased latency to fall from the rotarod on Day 1 of testing compared to WT and GluA2G/G mice, however, no differences were observed between any of the genotypes on testing days 2 and 3 indicating a normal motor learning ability for all groups across the testing period (RM ANOVA for time: F(2,232) = 15.16, p < 0.0001; for genotype: F(3,116) = 4.96, p < 0.01; n’s: WT = 33, GluA2G/G = 24, J20 = 36, GluA2G/G/J20 = 27). Each value represents the mean ± the SD for bar graphs and SEM for line graphs. *p < 0.05, **p < 0.01 ***p < 0.001, ****p < 0.0001. [file 13024_2023_632_MOESM8_ESM.tiff]
